# Shared and distinct interactions of type 1 and type 2 Epstein-Barr Nuclear Antigen 2 with the human genome

**DOI:** 10.1186/s12864-024-10183-8

**Published:** 2024-03-12

**Authors:** Kenyatta C. M. F. Viel, Sreeja Parameswaran, Omer A. Donmez, Carmy R. Forney, Matthew R. Hass, Cailing Yin, Sydney H. Jones, Hayley K. Prosser, Arame A. Diouf, Olivia E. Gittens, Lee E. Edsall, Xiaoting Chen, Hope Rowden, Katelyn A. Dunn, Rui Guo, Andrew VonHandorf, Merrin Man Long Leong, Kevin Ernst, Kenneth M. Kaufman, Lucinda P. Lawson, Ben Gewurz, Bo Zhao, Leah C. Kottyan, Matthew T. Weirauch

**Affiliations:** 1 Molecular and Developmental Biology Graduate Program, University of Cincinnati College of Medicine, Cincinnati, OH 45267 USA; 2 Center for Autoimmune Genomics and Etiology, Cincinnati Children’s Hospital Medical Center, Cincinnati, OH 45229 USA; 3 Department of Molecular Biology and Microbiology, Tufts University School of Medicine, 145 Harrison Ave, Boston, MA 02111 USA; 4 Division of Infectious Diseases, Department of Medicine, Brigham and Women’s Hospital, Harvard Medical School, Boston, MA 02115 USA; 5 Department of Pediatrics, University of Cincinnati College of Medicine, Cincinnati, OH 45267 USA; 6 Division of Human Genetics, Cincinnati Children’s Hospital Medical Center, Cincinnati, OH 45229 USA; 7 Division of Biomedical Informatics, Cincinnati Children’s Hospital Medical Center, Cincinnati, OH 45229 USA; 8 Division of Developmental Biology, Cincinnati Children’s Hospital Medical Center, Cincinnati, OH 45229 USA

**Keywords:** Autoimmune disorders, ChIP-seq, EBNA2, EBV, Functional genomics, Gene regulation, Genetic risk loci, Transcription factors, Viruses

## Abstract

**Background:**

There are two major genetic types of Epstein-Barr Virus (EBV): type 1 (EBV-1) and type 2 (EBV-2). EBV functions by manipulating gene expression in host B cells, using virus-encoded gene regulatory proteins including Epstein-Barr Nuclear Antigen 2 (EBNA2). While type 1 EBNA2 is known to interact with human transcription factors (hTFs) such as RBPJ, EBF1, and SPI1 (PU.1), type 2 EBNA2 shares only ~ 50% amino acid identity with type 1 and thus may have distinct binding partners, human genome binding locations, and functions.

**Results:**

In this study, we examined genome-wide EBNA2 binding in EBV-1 and EBV-2 transformed human B cells to identify shared and unique EBNA2 interactions with the human genome, revealing thousands of type-specific EBNA2 ChIP-seq peaks. Computational predictions based on hTF motifs and subsequent ChIP-seq experiments revealed that both type 1 and 2 EBNA2 co-occupy the genome with SPI1 and AP-1 (BATF and JUNB) hTFs. However, type 1 EBNA2 showed preferential co-occupancy with EBF1, and type 2 EBNA2 preferred RBPJ. These differences in hTF co-occupancy revealed possible mechanisms underlying type-specific gene expression of known EBNA2 human target genes: *MYC* (shared), *CXCR7* (type 1 specific), and *CD21* (type 2 specific). Both type 1 and 2 EBNA2 binding events were enriched at systemic lupus erythematosus (SLE) and multiple sclerosis (MS) risk loci, while primary biliary cholangitis (PBC) risk loci were specifically enriched for type 2 peaks.

**Conclusions:**

This study reveals extensive type-specific EBNA2 interactions with the human genome, possible differences in EBNA2 interaction partners, and a possible new role for type 2 EBNA2 in autoimmune disorders. Our results highlight the importance of considering EBV type in the control of human gene expression and disease-related investigations.

**Supplementary Information:**

The online version contains supplementary material available at 10.1186/s12864-024-10183-8.

## Background

Epstein-Barr Virus (EBV) is a human gammaherpesvirus that infects over 90% of the world’s population [1]. While usually asymptomatic, acute EBV infection can cause infectious mononucleosis. After initial infection, the virus enters a latent infection of B lymphocytes and persists throughout the lifetime of the host [2]. EBV is associated with a number of human cancers (e.g., Hodgkin's lymphoma [3], Burkitt lymphoma [2], and nasopharyngeal carcinoma [4]) and autoimmune disorders (e.g., systemic lupus erythematosus (SLE) and multiple sclerosis (MS)). The causal role of EBV in autoimmune disease has become clearer with recent molecular and epidemiological studies [5–9]. For example, a recent study reported a 32-fold increase in MS risk following EBV infection and showed that MS flare ups occurred after an increase of EBV protein levels in the serum [10]. Given the myriad of diseases associated with EBV infection, understanding the molecular mechanisms by which EBV triggers human disease is critical for the development of improved preventative strategies and therapeutic interventions.

Two distinct genetic types of EBV exist: EBV type 1 (EBV-1) and EBV type 2 (EBV-2) [11–13]. Our knowledge of the virus is primarily based on EBV-1, first characterized in the 1960s, while EBV-2 is less well studied [14]. EBV-1 is classified as a global virus, while most literature describes EBV-2 as being endemic to Africa [15], although evidence suggests it is likely much more widespread than previously thought. For example, approximately 20% of college students in England between 1999 and 2000 tested seropositive for EBV-2 [16, 17]. Similarly, 90% of adult patients with MS in Spain were coinfected with both EBV-1 and EBV-2 [18]. EBV type 2 has been detected in oral swabs of pediatric patients with MS in Canada [19], and a South Korean study found that 54.5% of patients with SLE were infected with both EBV types 1 and 2 [20]. Collectively, these findings indicate that EBV-2 can be found globally, and it may therefore be more relevant to global human disease than previously thought.

EBV-1 and EBV-2 both express forms of Epstein-Barr Nuclear Antigen 2 (EBNA2), a viral transactivator protein that impacts gene expression in the host and viral genomes [21–23]. EBNA2 is typically expressed as part of the viral latency III program, which transforms primary human B cells into immortalized lymphoblastoid cell lines (LCLs) [24, 25]. EBV-1 and EBV-2 EBNA2 differ substantially at the sequence level, sharing only 53% amino acid identity between the EBV-1 B95.8 strain and the EBV-2 AG876 strain [12]. Functionally, EBNA2 types 1 and 2 have unique effects on EBV type-specific primary human B cell transformation [26] and growth [27]. Point mutations within both the transactivation domain and casein kinase II phosphorylation site of EBNA2 type 2 drastically alter the ability of EBV-2 to maintain LCL growth in vitro [26, 27].

Type 1 EBNA2 has been implicated in several autoimmune diseases [8, 9, 28–30], with nearly half of known SLE and MS risk loci occupied by EBNA2 [5]. Furthermore, EBNA2 interacts with many of these risk loci in a genotype-dependent manner, suggesting that variation in the host genome impacts binding of EBNA2 to risk variants [5]. EBNA2 also substantially rewires the human gene regulatory network in infected B cells, and these EBNA2-dependent chromatin-altering events are highly enriched at autoimmune risk loci [9].

EBNA2 lacks a DNA binding domain. Instead, it interacts with human transcription factors (hTFs) to bind DNA and regulate expression. A number of transcription factors are known cofactors of EBNA2: recombination signal binding protein for immunoglobulin kappa J region (RBPJ) [22, 31], spleen focus forming virus proviral integration oncogene (SPI1, also referred to as PU.1) [32, 33], and Early B Cell Factor 1 (EBF1) [34, 35]. While much is known based on EBV-1 infected cells and the associated EBNA2 type 1 proteins, the substantial amino acid differences with EBNA2 type 2 suggest that the interaction of these strains with both the host genome and hTF partners may vary between types. 

**Fig. 1:**
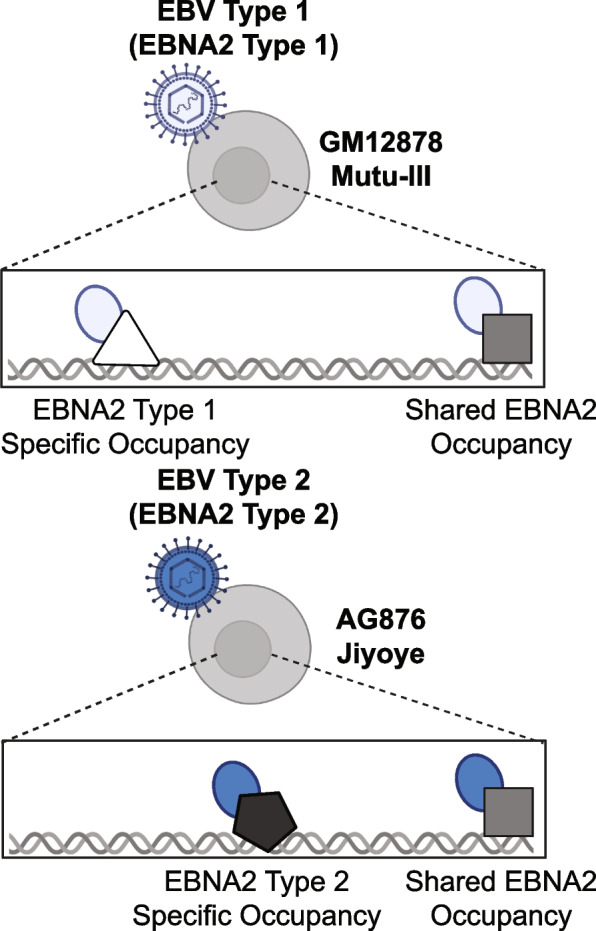
Schematic overview: study design for identification of type-specific EBNA2 binding events and binding partners. Starting with two human B cell lines with type 1 EBV infection and two human B cell lines with type 2 EBV infection, we performed EBNA2 chromatin immunoprecipitation (ChIP-seq). Using these data, we identified shared and type-specific EBNA2 ChIP-seq peaks in the human genome. Differential EBNA2 interactions with human cofactors were predicted computationally and validated experimentally

In this study, we systematically examine both EBV-1 and EBV-2 EBNA2 genomic binding events, interaction partners, and disease genetic risk locus occupancy to determine both shared and unique functions (Fig. 1). Using a series of functional genomic experiments, we identify similarities and differences in EBNA2 type 1 and 2 genomic binding locations and interacting DNA binding partners. Furthermore, we identify shared and unique associations between types 1 and 2 EBNA2 and loci associated with human disease risk. Collectively, these results greatly expand our understanding of type 2 EBNA2 function and potential disease roles.

## Results

### Extensive type-specific EBNA2 binding in the human genome

To our knowledge, no prior study has compared EBNA2 type 1 and type 2 binding on a genome-wide scale. To this end, we used ChIP-seq to systematically establish where EBNA2 types 1 and 2 bind within the human genome (Fig. 1). We performed EBNA2 type 2 ChIP-seq experiments in the B cell line AG876 and in the B cell line Jiyoye, each of which are infected by EBV-2. Likewise, we performed EBNA2 type 1 ChIP-seq experiments in the LCL GM12878 (EBV-1). We supplemented these data with publicly available Mutu-III (EBV-1) data [36] (Supplemental Dataset 1). The majority of these datasets were of excellent quality based on ChIP-seq ENCODE standards (see Methods). Specifically, all EBNA2 datasets had over 3,000 peaks, with Fraction of Reads in Peaks (FRiP) scores ranging from 0.04 – 0.35 (Supplemental Dataset 2). As expected, all EBNA2 datasets had strong motif enrichment results for known cofactors (RBPJ or EBF1 motifs ranked #1 in every experiment, with *p*-values < 10^–300^) as well as strong overlap with public ChIP-seq datasets (EBNA2 or RBPJ ChIP-seq from EBV infected B cells ranked #1 in every experiment, with enrichment *p*-values < 10^–210^) (Supplemental Datasets 3—4).

**Fig. 2:**
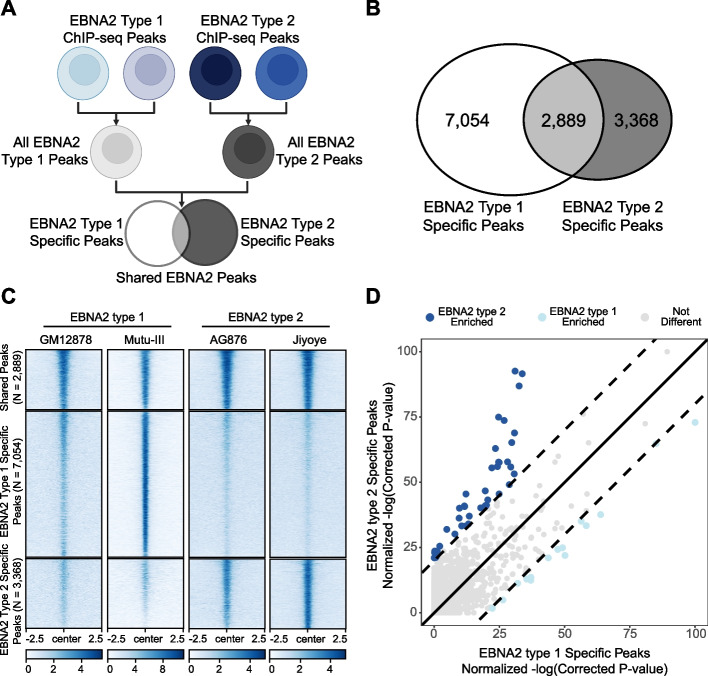
EBNA2 types 1 and 2 interact with the human genome in a type-specific manner. **A** Schematic for identification of type 1 and type 2 specific EBNA2 peaks. **B** Shared and type-specific EBNA2 peak counts. **C** ChIP-seq signal strength (normalized read depth) for type 1 (GM12878, Mutu-III) and type 2 (AG876, Jiyoye) EBNA2 at shared and type-specific regions. As expected, shared peaks (top) have equivalent signal strength between the four cell lines. EBNA2 type 1 specific peaks have greater signal strength in EBV-1 cell lines (middle left) compared to EBV-2 cell lines (middle right). Likewise for type 2 (bottom). See Methods for EBNA2 ChIP-seq analysis details. **D** Identification of EBNA2 type-specific enrichment of Gene Ontology Biological Processes. GO enrichment analysis of Biological Processes was performed within EBNA2 type 1 specific and type 2 specific ChIP-seq peaks. Each dot represents the normalized significance of one GO term. Type 1 specific (x-axis) and type 2 specific (y-axis) normalized significance are compared. The solid black line indicates equivalent significance between the compared peak sets. Dashed lines indicate the cut off for type specific enrichment (difference of 20% or more)

We first compared the peak locations between each EBNA2 ChIP-seq dataset (Fig. 2A), revealing 2,889 shared peaks, 3,368 peaks unique to EBNA2 type 2, and 7,054 peaks unique to EBNA2 type 1 (Fig. 2B). As expected, we found equal signal strength (normalized read depth) within the shared peaks across all four cell lines (AG876, Jiyoye, Mutu-III, and GM12878), stronger type 1 signal in type 1 specific peaks, and stronger type 2 signal in type 2 specific peaks (Fig. 2C, top, middle, and bottom groups, respectively). To evaluate the robustness of our type-specific findings, we created “type 1 specific stringent peaks” and “type 2 specific stringent peaks” to complement the “lenient peaks” presented throughout this study (see Methods). Using these stringent EBNA2 ChIP-seq peak datasets, we identified 796 shared peaks, 632 peaks unique to EBNA2 type 2, and 551 peaks unique to EBNA2 type 1 (Supplemental Figure 1A). Collectively, these data reveal the existence of both shared and distinct genomic binding locations for EBNA2 types 1 and 2.

Next, to identify pathways that might be differentially regulated by type 1 and type 2 EBNA2, we used the GREAT software package [37] to perform gene set enrichment on genes located near EBNA2 binding sites for the gene ontology (GO) biological process category. To determine which GO terms were impacted by EBNA2 types, we defined unequal enrichment when the difference in the normalized FDR between type-specific peaks was greater than 20% (Fig. 2D). While most GO terms were considered “equally enriched” between EBNA2 type 1 and EBNA2 type 2, 16 pathways had higher enrichment towards type 1 and 41 had higher enrichment in type 2 (Supplemental Dataset 5). All enriched biological pathways were relevant to immune cells. Type 1 specific EBNA2 peaks were generally enriched for pathways related to the immune response. EBNA2 type 1 peaks were specifically enriched for the Fc Receptor pathway (difference of over 20 orders of magnitude in the adjusted *p*-value). Notably, there is increased expression of *FCGR* in EBV-1 transformed cells compared to EBV-2 transformed cells in our RNA-seq data. Four out of the 41 EBNA2 type 2 enriched GO terms were related to T cell activation, proliferation, and differentiation. These T cell-specific findings are consistent with the unique ability of EBV-2 to infect T cells [38, 39]. EBNA2 type 2 specific peaks are also enriched for pathways related to cell death, which is consistent with the reduced ability of type 2 EBV to transform B cells relative to type 1 EBV [38, 40].

To determine whether differences in chromatin accessibility could account for the observed differences in EBNA2 binding, we next measured chromatin accessibility in EBV type 1 or EBV type 2 infected B cells. To this end, we also generated ATAC-seq data in the AG876, Jiyoye, GM12878, and Mutu-III cell lines. All datasets were of ‘Excellent’ or ‘Good’ quality (see Methods). For example, all datasets had over 40,000 peaks, with FRiP scores greater than 0.12 and Transcription Start Sites (TSS) enrichment scores greater than 7 (average of 16.9) (Supplemental Dataset 2 ).

We next re-focused our analysis on the 31,379 regions in the genome with shared accessibility across the four cell lines (Supplemental Figure 2 and see Methods). 22.3% of genomic regions occupied by EBNA2 in at least one of the four cell lines were located within these commonly accessible loci. Within these commonly accessible loci, we found 1,366 EBNA2 type 2 specific binding locations, 3,405 EBNA2 type 1 specific binding locations, and 2,234 EBNA2 locations shared across type 1 and 2 (Supplemental Figure 3). Notably, the proportions of these groups are nearly identical to the proportions we observed before accounting for chromatin accessibility differences (Fig. 2B). Thus, type-specific EBNA2 binding events cannot simply be accounted for by differences in chromatin accessibility across cell lines.

### Shared and distinct co-occupancy of human TFs with EBNA2 types 1 and 2

**Fig. 3:**
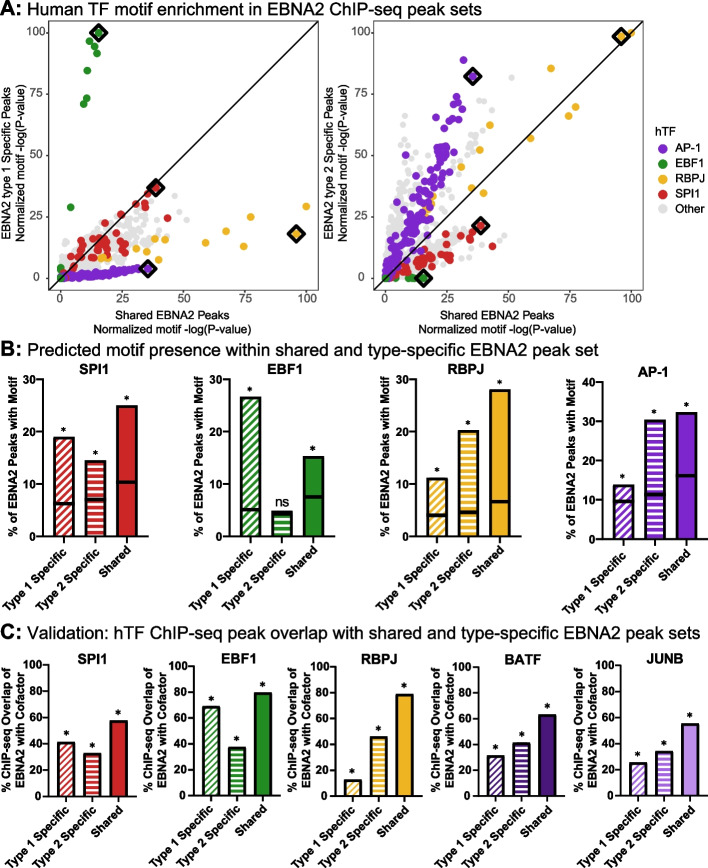
Identification of shared and type-dependent EBNA2 human cofactors. **A** Unbiased computational prediction of EBNA2 human cofactors. Human transcription factor (hTF) motif enrichment analysis was performed within EBNA2 type 1 specific, type 2 specific, and shared peaks. Each dot represents the normalized significance of one hTF motif. Type 1 specific (y-axis, left) or type 2 specific (y-axis, right) normalized motif significance is compared to shared peaks (x-axis in both panels). Black diamonds indicate exemplar hTF motifs for the four hTF classes that are depicted in (**B**). The black line indicates equivalent significance between the compared peak sets. Motifs are colored by class. **B** Frequency of occurrence of exemplary motifs in EBNA2 shared and type-specific peak sets. For the four exemplary motifs, the percent of peaks containing predicted binding sites for the motif is shown. Each bar represents percent foreground (i.e., the percent of actual peak DNA sequences containing a match to the motif). The horizontal black line within each bar depicts percent background (i.e., the percent of randomly selected genome sequences, matching GC content). Asterisks indicate significant motif enrichment (*P* < 0.05), as calculated by HOMER. **C** Experimental validation of predicted EBNA2 co-occupancy with hTFs. Co-occupancy was assessed by hTF and EBNA2 ChIP-seq peak overlap. The hTFs BATF and JUNB were chosen as representative AP-1 family members (see Results). For each of the five hTFs, a union peak set was created by combining peaks across all cell lines. For each bar, the percent of each hTF union peak set overlapping each EBNA2 ChIP-seq peak category is shown. Datasets with significant overlap between EBNA2 peak sets and the union peak set of hTFs (as calculated by RELI) are indicated with asterisks (*P* < 0.05). Note the consistency between the motif-based predictions (**B**) and ChIP-seq experimental validation results (**C**)

EBNA2 lacks a DNA binding domain and requires hTF partners to occupy genomic targets. We hypothesized that the DNA sequences located under EBNA2 ChIP-seq peaks might offer insight into shared and type-specific preferences of EBNA2 for hTF binding partners. To this end, we performed unbiased human transcription factor binding site motif enrichment analysis at shared and type-specific EBNA2 regions. As expected, the most strongly enriched motif classes for both types of EBNA2 include SPI1, RBPJ, and EBF1 (Fig. 3A). SPI1 motifs are equivalently enriched in shared, type 1, and type 2 specific peaks (Fig. 3A, red dots near diagonal lines of both panels). RBPJ motifs were enriched in the shared peaks, with equivalent enrichment in type 2 specific peaks and somewhat less enrichment in type 1 specific peaks (Fig. 3A, right panel, yellow dots along the diagonal line; left panel, yellow dots near x-axis, respectively). Strikingly, we observed a very strong preference for EBF1 motifs at type 1 specific regions compared to those that are shared (Fig. 3A, left panel, green dots near y-axis). Likewise, we observed a strong preference for AP-1 motifs (which are highly present at the enhancers of many cell types [41–44]) at type 2 specific regions compared to those at shared peaks (Fig. 3A, right panel, purple dots near y-axis).

Focusing on these four classes of motifs, we next examined motif presence (as opposed to enrichment) at shared and type-specific EBNA2 regions. Comparing type-specific motif presence allows us to further classify potential type-specific EBNA2 hTF preferences. For each class of motifs, we identified an exemplary motif (Fig. 3A, boxed motifs) and calculated the motif presence frequency within shared and type-specific EBNA2 peak sets. For all four hTFs, 15–32% of the shared EBNA2 peaks overlap their respective motifs (i.e., 15–32% of the underlying DNA sequences contain strong predicted binding sites for the corresponding hTF based on its exemplary motif) (Fig. 3B, solid bars). We classify an hTF preference as *equivalent* when 1) the motif is enriched in both type 1 and type 2 peaks and 2) there is less than a 5% difference in motif overlap in type-specific EBNA2 peak sets. Using these criteria, EBNA2 type 1 and 2 have an equivalent preference for SPI1 (Fig. 3B: 19% vs 15%). In contrast, RBPJ and AP-1 motif analyses predict an EBNA2 type 2 specific preference, and EBF1 analyses predict a type 1 specific preference (Fig. 3B). Identical predictions were made based upon motif enrichment statistics (Supplemental Figure 4).

To test these predictions, we performed ChIP-seq experiments for RBPJ, EBF1, and SPI1 in the GM12878, Jiyoye, and AG876 cell lines. BATF and JUNB were chosen as representative AP-1 hTFs based on their expression levels across the cell lines. We supplemented these 20 new experiments with publicly available data (see Methods). All hTF ChIP-seq datasets were of high quality and met or exceeded ENCODE data quality standards. Datasets had a peak count ranging from 2,061 (RBPJ) to 48,570 (SPI1), with FRiP scores ranging from 0.04 – 0.35 (Supplemental Dataset 2 ). Each dataset had strong motif enrichment results and statistically significant overlap with public ChIP-seq datasets of the same protein (Supplemental Datasets 3-4).

These ChIP-seq experiments confirmed co-occupancy of each of these factors at regions bound by EBNA2 type 1 and 2. In particular, shared EBNA2 peaks had 58%, 80%, 79%, 63%, and 56% overlap with SPI1, EBF1, RBPJ, BATF, and JUNB ChIP-seq peak sets, respectively (Fig. 3C). This overlap is consistent with previous observations focused on type 1 EBNA2 ChIP-seq [45]. Overall, the ChIP-seq co-occupancy results were highly consistent with the motif predictions (i.e., compare the patterns of like-colored bars in Fig. 3B and C). Similar to the motif analysis, we classified type 1 and type 2 EBNA2 as having *equivalent* preferences for an hTF if 1) there is statistically significant overlap of the type-specific EBNA2 peaks and hTF peaks and 2) the percent overlap difference is less than 10%. As predicted by the motif analysis, types 1 and 2 EBNA2 had equivalent preference for SPI1 (Fig. 3C, left). We also confirmed a strong type 1 preference for EBF1 (31.5% difference). Likewise, we confirmed a type 2 EBNA2 preference for RBPJ (33.4% difference). Contrary to our predictions, BATF and JUNB equivalently occupied loci with type 1 and type 2 EBNA2 based on our criteria. However, when we focused on regions of shared open chromatin between EBV types, the results meet our criteria (Supplemental Figure 5). Notably, examination of the stringent type 1 and type 2 specific EBNA2 peak sets revealed the same hTFs that were predicted using our “lenient” peak sets (Supplemental Figure 1B).

### Biochemical assessment of differential EBNA2 type 1 and 2 preferences for RBPJ and EBF1

To assess the biochemical basis for type-specific EBNA2 co-occupancy with RBPJ across the genome, we next performed co-immunoprecipitation (Co-IP) experiments to test the hypothesis that EBNA2 type-specific ChIP-seq peak colocalization patterns reflect differential physical interaction preferences with RBPJ. We performed Co-IP using nuclear lysates from the Akata (EBV negative), GM12878 (EBV-1), and AG876 (EBV-2) cell lines (Supplemental Figure 6). Co-immunoprecipitation confirmed that both type 1 and type 2 EBNA2 immunoprecipitated RBPJ (Supplemental Figure 6). In our experiments, we did not identify notable differences in the ability of type 1 and type 2 to Co-IP RBPJ. Thus, we conclude that type-specific RBPJ binding of EBNA2 is not based on differences in the biochemical interaction of the proteins. As in previous studies [22], we found little evidence of direct EBNA2-EBF1 or EBNA2-SPI1 interactions through Co-IP experiments. We therefore conclude that EBNA2 type 1 and type 2 likely interact with EBF1 and SPI1 through intermediate proteins and not through direct biochemical interactions.

To further understand the EBNA2 type 1 preference for EBF1, we measured EBF1 expression across the EBV type 1 and type 2 cell lines. RNA was extracted from the AG876, Jiyoye, and GM12878 cell lines. We also included Mutu-III RNA-seq data from our previous study [46]. The resulting data were of high quality (Quality Control (QC) Report, Supplemental Dataset 2) and displayed strong agreement between replicates (Supplemental Figure 7). For transcriptomic analyses, we calculated the average normalized counts (using DESeq2, see Methods) of EBNA2 and each hTF for each EBV type (EBV-1 or EBV-2). The relative expression was compared to the protein level expression of EBV-1 infected (GM12878 and Raji) and EBV-2 infected (AG876 and Jiyoye) cells using Western blots (see Methods). When the average relative expression between type 1 and type 2 is over two, we conclude that there is type-specific expression.

EBV type 1 cells demonstrated strong type-specific expression of *EBF1*, with 8.5-fold higher levels of *EBF1* in type 1 cells relative to EBV type 2 cell lines (Jiyoye average normalized count = 1, AG876 average normalized count = 401) (Table 1). Type 1 elevated EBF1 expression was also found at the protein level, with 5.4-fold greater EBF1 expression in EBV-1 cell lines compared to EBV-2 cell lines (Supplemental Figure 8, Table 2). These data indicate that EBF1 is not available to co-occupy genomic regions with EBNA2 in Jiyoye cells, which explains the observed lack of enrichment of EBF1 motifs in EBNA2 Jiyoye ChIP-seq peaks.

**Table 1:** Expression levels (normalized RNA-seq read counts) for genes of interest. Values are the mean of biological triplicates. N/A Not applicable

	**BATF**	**JUNB**	**SPI1**	**EBF1**	**RBPJ**	**EBNA2 TYPE 1 (B95.8)**	**EBNA2 TYPE 2 (AG876)**
GM12878	1,131	5,887	998	2,784	5,572	10,009	N/A
MUTU-III	238	1,751	1,142	650	3,841	3,359	N/A
AG876	1,407	3,661	826	401	8,285	N/A	5,019
JIYOYE	1,259	6,607	515	1	10,795	N/A	10,651
RAMOS (EBV-)	77	935	824	979	3,336	N/A	N/A

**Table 2:** Average Western blot densitometry signal for proteins of interest in this study. Values are the mean of biological triplicates

	**BATF**	**JUNB**	**SPI1**	**EBF1**	**RBPJ**	**EBNA2**
GM12878	885	3960	343	219	2170	68
RAJI	396	1560	775	339	1930	61
AG876	671	1750	292	94	2970	76
JIYOYE	718	1580	112	9	1800	45

Because Jiyoye cells do not express EBF1, we next performed an additional set of analyses focused on comparing AG876 and GM12878 as representative EBV-2 and EBV-1 cell lines, respectively. After limiting the analysis to AG876, type-specific EBF1 expression differences were mirrored in the motif enrichment and ChIP-seq overlap analyses: we identified a greater proportion of peaks with the EBF1 motif in GM12878 (type 1) compared to AG876 (type 2) (Table 3). Consistently, EBF1 overlapped GM12878 EBNA2 ChIP-seq peaks at a higher percentage than AG876 EBNA2 ChIP-seq peaks (78% and 67%, respectively; Supplemental Figure 9). These findings indicate that while EBNA2 type 1 prefers EBF1 compared to EBNA2 type 2, EBNA2 type 2 can likely also interact with EBF1 on a more limited basis.

**Table 3:** EBF1 motif enrichment across datasets. Results are ordered from most to least enriched

*EBNA2 Peaks*	*% of Target Sequences with Motif*	*% of Background Sequences with Motif*	*p****-value***	*Enrichment*
***Type 1 Specific Peaks***	26.7%	5.1%	10^–774^	5.2
*Mutu-III*	24.7%	5.7%	10^–731^	4.3
*GM12878*	16.2%	4.2%	10^–139^	3.9
*Shared EBNA2 Peaks*	15.3%	7.5%	10^–44^	2.0
*AG876*	10.5%	5.6%	10^–27^	1.9
*Jiyoye*	7.2%	5.3%	10^–8^	1.4
***Type 2 Specific Peaks***	4.5%	4.6%	NS	N/A

*NS *Not significant, *N/A *Not applicable

To further dissect the enhanced co-binding of EBNA2 type 1 with EBF1 relative to EBNA2 type 2, we performed split nanoluciferase experiments (Supplemental Figure 10). For these experiments, we cloned one half (small bit, SmBIT) of the nanoluciferase molecule to EBF1 and the other half (large bit, LgBIT) to EBNA2. In this experimental system, the detection of luciferase is dependent upon the two molecules being proximal to one another (i.e., SmBIT and LgBIT come together to allow nanoluciferase to produce light). We found that EBNA2 type 2 and EBF1 are in close proximity to each other at a higher frequency than EBNA2 type 1 and EBF1. These findings suggest that even if EBNA2 is not biochemically interacting with EBF1, the proteins are co-localizing in both type 1 and type 2 EBV cells. These results are consistent with the conclusion that type 1 enhanced co-binding with EBF1 is due to lower expression of EBF1 in cells that express type 2 EBNA2.

### EBNA2 type 1 and type 2 binding at known EBNA2 target genes

Previous studies have firmly established EBNA2 target genes that are important for both the virus and host [27, 40, 47–52]. The current study offers a new opportunity to assess shared and distinct EBNA2 type 1 and 2 occupancy at these EBNA2-controlled loci. We broadly hypothesized that type-shared and type-specific EBNA2 occupancy patterns will follow the type-specific expression levels of EBNA2 target genes. We again categorized genes as having type-specific expression when the average expression (normalized counts calculated by DESeq2) difference between the EBNA2 types is more than two.

**Fig. 4:**
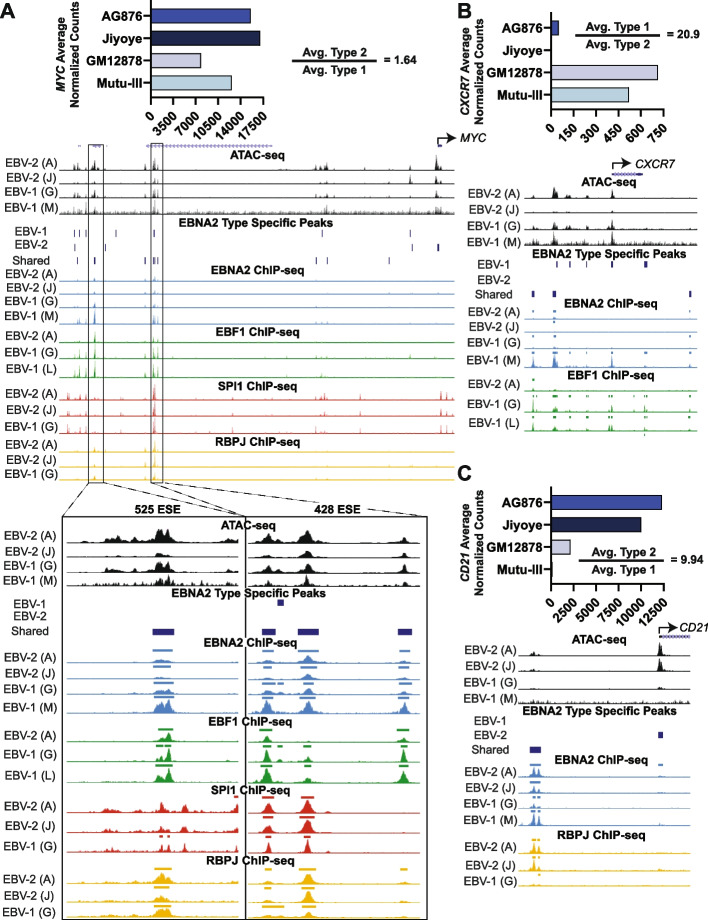
Type-specific expression of human genes that corresponds with type-specific EBNA2 and hTF partner genomic occupancy. One example is shown for each of the following: shared EBNA2 binding (**A**: *MYC*, chr8:128,168,540–128,775,448), type 1 specific EBNA2 binding (**B**: *CXCR7*, chr2:237,441,986–237,513,677), and type 2 specific EBNA2 binding (**C**: *CD21*, chr1:207,601,245–207,633,439). For each locus, the normalized counts (DESeq2) of the human gene are shown above a UCSC Genome Browser screenshot depicting (top to bottom): the gene, chromatin accessibility, EBNA2 binding (ChIP-seq), and hTF binding (ChIP-seq) in each cell line. Type-specific and shared EBNA2 peaks are indicated above the EBNA2 ChIP-seq tracks. In panel A, previously identified type 1 EBNA2 super enhancers at the *MYC* locus ([57]; *MYC* ESE2, chr8:128,312,176–128,320,865; *MYC* ESE1: chr8:128,215,588–128,228,144) are boxed and labeled. Data from biological replicates are shown throughout. A = AG876; J = Jiyoye; M = Mutu-III; G = GM12878; L = LCL. Data ranges: *MYC* Overview [ATAC-seq (0 to 5; Mutu-III 0 to 0.7); EBNA2 ChIP-seq (0 to 14); EBF1 ChIP-seq (0 to 5); SPI1 ChIP-seq (0 to 5); RBPJ ChIP-seq (0 to 5)]; *MYC* 525 ESE [ATAC-seq (0 to 3; Mutu-III 0 to 0.4); EBNA2 ChIP-seq (0 to 13); EBF1 ChIP-seq (0 to 5); SPI1 ChIP-seq (0 to 1); RBPJ ChIP-seq (0 to 2)]; *MYC* 428 ESE [ATAC-seq (0 to 4; Mutu-III 0 to 0.7); EBNA2 ChIP-seq (0 to 8); EBF1 ChIP-seq (0 to 4); SPI1 ChIP-seq (0 to 5); RBPJ ChIP-seq (0 to 5)]; CXCR7 [ATAC-seq (0 to 3; Mutu-III 0 to 0.4); EBNA2 ChIP-seq (0 to 10); EBF1 ChIP-seq (0 to 4)]; *CD21* [ATAC-seq (0 to 8; Mutu-III 0 to 0.4); EBNA2 ChIP-seq (0 to 6); SPI1 ChIP-seq (0 to 3); RBPJ ChIP-seq (0 to 3)]

Based on studies focused on both type 1 and 2 EBV, EBNA2 upregulates *MYC,* resulting in accelerated B cell proliferation and immortalization [40, 47, 48]. In our data, we observed high expression of *MYC* across all four cell lines, with an average normalized count difference of only 1.64 (Fig. 4A, top panel). We observe higher *MYC* expression in Mutu-III, AG876, and Jiyoye cell lines. These three cell lines are derived from Burkitt lymphomas, where *MYC* translocation drives *MYC* expression [53–55]. Two well characterized “EBNA2 Super Enhancers” (ESE) are located approximately 520 kb (the “525 ESE”) and 430 kb (the “428 ESE”) upstream of *MYC*, each of which control *MYC* expression in infected B cells [45, 56, 57]. Consistently, we identified strong conservation of EBNA2 peaks between all four cell lines at both ESE sites (Fig. 4A, bottom panel). We also observed robust binding of EBNA2 co-partners EBF1, SPI1, and RBPJ at the same regions in both type 1 and type 2 EBV infected cell lines. We conclude that the EBNA2-based gene regulatory mechanisms underlying its control of *MYC* expression are largely conserved across EBV types.

Numerous studies have established that EBNA2 type 1 drives higher expression of Chemokine (C-X-C Motif) Receptor 7 (*CXCR7*; also referred to as *ACKR3*) compared to EBNA2 type 2 [27, 40, 49]. Our expression data confirm these results, with a 20.9-fold increase in average *CXCR7* expression in EBV-1 cell lines compared to EBV-2 cell lines (Fig. 4B, top panel). Correspondingly, we observed five type 1 specific and no type 2 specific EBNA2 ChIP-seq peaks in the *CXCR7* locus (Fig. 4B, bottom panel). Little is known regarding which co-partners regulate *CXCR7* along with EBNA2. While we observed equal RBPJ and SPI1 binding activity in the GM12878, Jiyoye, and AG876 cell lines, we observed stronger EBF1 binding activity in GM12878 compared to AG876 at the type 1 EBNA2 specific binding events. Thus, type 1 specific binding of EBNA2 and EBF1 are consistent with the observed higher expression levels of *CXCR7* in EBV-1 infected cell lines. To further explore EBF1 colocalization with EBNA2, we performed ChIP-qPCR for EBF1 at three EBNA2 ChIP-seq peaks in the *CXCR7* locus (Supplemental Figure 11). As expected, we found that EBF1 had much stronger binding within EBNA2 peaks in type 1 cells compared to type 2 cells, including at the type 1 EBNA2 specific + 3 kb region.

For EBV to enter lymphocytes and epithelial cells, viral protein gp350 interacts with the human cluster of differentiation 21 (CD21) protein, which is also referred to as complement receptor 2 [39, 58–60]. Type 1 EBNA2 interacts with RBPJ to increase the expression of *CD21* [50–52]. To our knowledge, no studies have examined this mechanism in the context of type 2 EBNA2. In our data, EBV type 2 cells had 9.9-fold higher average *CD21* expression compared to EBV type 1 cells (Fig. 4C, top panel). Therefore, we hypothesized that there would be increased EBNA2 type 2 activity at the *CD21* locus compared to type 1. At the *CD21* locus, we observed two regions with type 2 specific accessibility: the promoter and 20 kb upstream (Fig. 4C, bottom panel). Chromatin was significantly more accessible in type 2 cell lines in both regions (DiffBind *p*-value = 1.12E-4). RBPJ also demonstrated enhanced co-occupancy at the 20 kB upstream EBNA2 peak in both type 2 cell lines relative to the type 1 cell line (DiffBind *p*-value = 5.37E-7). These mechanisms could explain the observed type 2 enhanced *CD21* expression levels.

### EBNA2 types 1 and 2 occupy shared and distinct human autoimmune disease risk loci

**Fig. 5:**
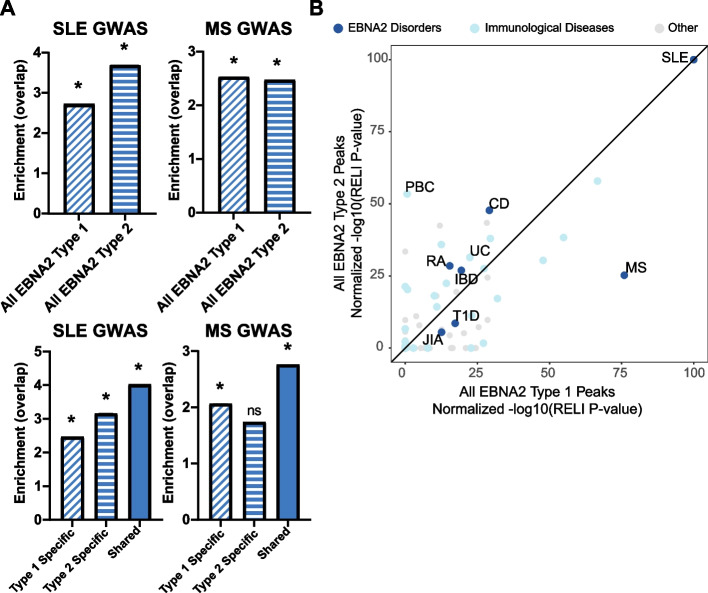
EBNA2 type 1 and 2 occupancy at human disease risk loci. Statistical enrichment of the overlap between type 1 and type 2 EBNA2 ChIP-seq peaks with human disease risk genetic variants. **A** Top: Enrichment of full EBNA2 ChIP-seq peak sets at disease risk loci for diseases previously established for type 1 EBNA2 (multiple sclerosis (MS) and systemic lupus erythematosus (SLE)). Bottom: Enrichment of type-specific and shared EBNA2 peak sets. Datasets with significant overlap (as calculated by RELI) are marked with asterisks (*P* < 0.05). **B** Enrichment of type-specific EBNA2 ChIP-seq peak sets at all disease risk loci. Each dot represents RELI results (normalized -log corrected *p*-value) for EBNA2 enrichment for a given disease. Results were normalized to the max negative log *p*-value for each EBNA2 dataset to facilitate comparisons. The black line indicates equivalently significant enrichment for type 1 and type 2 EBNA2. Previously established “EBNA2 disorders” (dark blue dots) and other diseases discussed in the text are labeled. Disease abbreviations: CD Celiac Disease, IBD Inflammatory Bowel Disease, JIA Juvenile Idiopathic Arthritis, MS Multiple Sclerosis, PBC Primary Biliary Cholangitis, RA Rheumatoid Arthritis, SLE Systemic Lupus Erythematosus, T1D Type 1 Diabetes, UC Ulcerative Colitis

We previously showed that EBNA2 type 1 (Mutu-III) ChIP-seq peaks directly overlap nearly half of the genetic risk loci for MS, SLE, and other autoimmune diseases (“EBNA2 disorders”) with robust and statistically significant enrichment according to our RELI algorithm [5, 9]. The present study provides the first opportunity to systematically identify possible connections between type 2 EBNA2 and human diseases. We first used RELI to assess the enrichment of EBNA2 type 1 and type 2 datasets from this study at MS and SLE disease risk loci. As expected, pooled type 1 EBNA2 peaks (GM12878 and Mutu-III) significantly overlapped the risk loci for both MS and SLE (Fig. 5A – diagonal striped bars). Importantly, we observed similar levels of enrichment for pooled type 2 EBNA2 peaks (AG876 and Jiyoye) (Fig. 5A – horizontal striped bars). To assess the peaks that are unique to type 1 and type 2 EBNA2, we next performed RELI using EBNA2 type 1 and type 2 specific peaks. We again observed statistically significant enrichment for type 1 and type 2 specific EBNA2 peaks at SLE disease risk loci. While EBNA2 type 1 specific peaks were statistically significant with an enrichment of 2.1 for MS risk loci, EBNA2 type 2 specific peaks were not significant (*p*-value = 1) (Fig. 5A, bottom graphs). Similar overall results are obtained using our stringent type-specific EBNA2 peaks (Supplemental Figure 1C). Taken together, these data indicate that shared and type-specific EBNA2 ChIP-seq peaks are highly enriched at MS and SLE genetic risk loci, indicating the likely importance of type 2 EBNA2 in these diseases in addition to type 1 EBNA2.

We next examined EBNA2 binding patterns at all disease risk loci contained in the Genome Wide Association Studies (GWAS) catalogue (see Methods, Supplemental Dataset 7). To this end, we compared disease risk loci enrichment between all pooled EBNA2 type 1 peaks and all EBNA2 type 2 peaks (Fig. 5B). We found that many of our previously reported EBNA2 disorders [5], such as systemic lupus erythematosus (SLE), inflammatory bowel disease (IBD) and celiac disease, are equivalently enriched between the two types of EBNA2 (Fig. 5B). In contrast, MS genetic risk loci are more significantly enriched for type 1 EBNA2 (Fig. 5B – bottom, right dot closer to x-axis). Meanwhile, risk loci for primary biliary cholangitis (PBC), which to our knowledge has never been connected to EBNA2, are more highly enriched for type 2 EBNA2 peaks (Fig. 5B – dot near y-axis), suggesting a new possible role for EBV-2 in this disease.

## Discussion

To our knowledge, no prior study has examined EBNA2 type 2 binding on a genome-wide scale. In this study, we examined EBNA2 interactions with the human genome, human TF partners, and human genetic risk loci in a virus type-specific manner. In addition to providing the first genome-wide type 2 EBNA2 ChIP-seq datasets, our findings show that (1) EBNA2 interacts with the human genome in a type-specific manner, (2) there is differential hTF interaction between EBNA2 types, (3) type-specific EBNA2 binding events are found proximal to genes regulated in an EBV type-specific manner, and (4) EBNA2 type 2 also interacts with autoimmune disease risk loci. Our findings show that, despite an overall sharing of cofactors for EBNA2 types 1 and 2, EBNA2 extensively interacts with the human genome at type-specific locations.

Our study builds upon previous studies that identified type and strain variations within EBV. Structurally, the low 53% sequence identity between EBNA2 type 1 and type 2 is striking, given the relatively high sequence similarity in other parts of the EBV genome [40]. Differences in EBNA2 type 1 and 2 gene expression levels have previously been shown to result in type-specific differences in human gene expression [13, 40]. Some of this type-specific human gene regulation is caused by a single amino acid at D442S that impacts binding at human genes, including *CXCR7* and *LMP1* [27]. In addition to variation in EBNA2, EBV type-specific variation within the *BZLF1* promoter plays a role in the ability of B cells to enter the lytic phase [14, 61–63]. Further, LCLs infected with type 2 viral strains have lower expression of IRF4 and EBF1 compared to LCLs infected with type 1 viral strains [63]. Similar findings were observed within our data, although *IRF4* did not meet our stringent criteria. These are only two of many known and currently unknown type-dependent differences.

Across all the human DNA bound by EBNA2, 78% was EBV type-specific, with only 22% shared across EBV types 1 and 2 (Fig. 2). Pathway-based analyses identified groups of genes near EBNA2 binding sites that were enriched for either type 1 or type 2 EBNA2. Pathways with type-specific enrichment were all related to the immune system – particularly, leukocyte biology and immune processes. For type 2 enriched pathways, it is notable that four T cell pathways are enriched along with three apoptotic pathways. These results align well with known biology of type 2 EBV where the type 2 strains are better able to infect T cells compared to the type 1 EBV virus [38–40]. Mechanistically, these differences in binding could be driven by the type-specific co-occupancy of EBNA2 with EBF1 (type 1) and RBPJ (type 2) (Fig. 3). Some of these differences in co-occupancy and hTF binding localize to genes with type-specific expression, such as *CXCR7* and *CD21* (Fig. 4). We previously demonstrated the impact of EBNA2 on human chromatin accessibility; however, differences in human genome binding and hTF co-occupancy were robust even after accounting for differences in chromatin accessibility (Supplemental Figures 3 and 5). Despite the magnitude of sequence differences, type 1 and 2 EBNA2 did retain some shared characteristics, including robust occupancy with SPI1 and AP-1 hTFs, an ability to interact with RBPJ and EBF1 (albeit to different, type-dependent degrees), and regulation of the previously described EBV super enhancer at the *MYC* locus [56] (Fig. 4A).

One of the important findings of this study is the identification of EBNA2 type 1 specific co-occupancy preference for EBF1. EBF1 is known to interact with EBNA2 at the N-terminal dimerization (END) domain [34]. When this domain is removed, EBNA2 can no longer interact with EBF1, and EBV fails to immortalize primary B cells due to their inability to complete the cell cycle. Our data suggest that EBNA2 type 1 has more EBNA2-EBF1 interactions compared to EBNA2 type 2. This provides a possible mechanistic basis for the more efficient immortalization of B cells by EBV-1 compared to EBV-2. The EBNA2 type 1 specific preference for EBF1 is particularly notable at the *CXCR7* locus (Fig. 4B). Functionally, the type-specific gene expression differences induced by EBNA2 could lead to type-specific cell immortalization, proliferation, and migration, particularly in inflammatory contexts.

We observed strong RBPJ motif enrichment and co-occupancy of RBPJ in genomic regions bound by both type 1 and type 2 EBNA2. These results are consistent with the importance of RBPJ as a cofactor for both types of EBNA2 [22, 64]. Additionally, our data support EBNA2 type 2 forming a greater number of unique RBPJ-EBNA2 complexes compared to EBNA2 type 1. For example, at *CD21* we identified enhanced RBPJ co-occupancy only with EBNA2 type 2. CD21 is the receptor that EBV uses to gain entry to human cells [50–52]. CD21 expression is increased in EBV type 2 cells, despite EBV type 2 having less ability to transform B cells. We hypothesize that type 2 enhanced *CD21* expression is a mechanism that EBV type 2 uses to attempt to augment its entry into cells.

Previous work established the clinical implications of type 1 EBNA2 binding to immune disease risk loci [5, 9]. This study expands these findings to include the possibility that EBNA2 type 2 interacts with a person’s individual risk variants to increase disease risk. EBV has been implicated in the complex etiology of multiple autoimmune diseases [10, 18, 30, 65–91]. However, most studies do not consider EBV type, designing experimental reagents that ambiguously detect EBNA2. Additionally, limited studies have assessed the potential for EBNA2 type 2 to contribute to autoimmune disease progression. In this study, we demonstrate an equivalent enrichment of type 2 EBNA2 binding at autoimmune genetic risk loci. These findings are consistent with epidemiological studies identifying EBV-2 in samples from patients with MS [18, 19]. Considering the global reach of EBV-1 and EBV-2 [18], future studies should consider additional mechanisms through which EBV-2 mediates the etiology of autoimmune diseases.

## Conclusions

Taken together, these findings provide the first genome wide assessment of EBNA2 types 1 and 2 in the context of genomic binding locations, human cofactors, and enrichment at human genetic risk loci. We identified both shared and distinct binding of EBNA2 type 1 and 2, with a type 1 preference for EBF1 and a type 2 preference for RBPJ. The binding of both type 1 and type 2 EBNA2 are enriched at autoimmune disease risk loci. Collectively, these data provide a foundation for future studies aimed at understanding type 2 EBNA2 biology and disease mechanisms.

## Methods

### Cell lines

GM12878, Mutu-III, and Raji cell lines were assessed as representative EBV-1 cell lines. GM12878 is an EBV-transformed LCL that is infected with the B95.8 virus. Mutu-III cells are Burkitt B cells infected with the EBV type 1 Mutu EBV virus that have the EBV latency III program. Raji cells are Burkitt B cells infected with the EBV type 1 Raji virus. Raji cells have several EBV genomic deletions, but they express most EBV latency III oncogenes. Jiyoye and AG876 (gifted from the Farrell Lab), infected with the EBV type 2 Jiyoye and EBV type 2 AG876 virus, respectively, were assessed as representative EBV-2 cell lines. The Akata cell line, which lacks EBV, was used for co-immunoprecipitation assays. The HEK-293 cell line, which also lacks EBV, was used for split nanoluciferase. All cell lines, except HEK-293 cells, were cultured in Roswell Park Memorial Institute (RPMI) 1640 (Dulbecco's Modified Eagle Medium (DMEM) was utilized for HEK-293 cells) with 2 mM L-glutamine, 0.2% Normocin, 10% fetal bovine serum, and 1X antibiotic–antimycotic at 200,000 – 500,000 viable cells/mL. Cells were incubated in vented flasks at 37 °C with 5% carbon dioxide in an upright position (flat for HEK-293 cells).

Supplemental Dataset 1 details all datasets used for analysis, which were processed using the computational tools detailed below.

### Chromatin Immunoprecipitation (ChIP) with sequencing (ChIP-seq)

ChIP-seq experiments were performed to identify locations in the human genome occupied by the two EBNA2 types and hTFs predicted to colocalize with EBNA2 in the EBV-2 and EBV-1 infected B cell lines. Specifically, for EBV-2, we generated new ChIP-seq datasets for EBNA2, BATF, EBF1, JUNB, RBPJ, and SPI1 in both the AG876 and Jiyoye cell lines. For EBV-1, we generated new ChIP-seq datasets for EBNA2 and RBPJ for the GM12878 cell line. We obtained publicly available GM12878 ChIP-seq datasets for BATF, EBF1, JUNB, and SPI1, and Mutu-III EBNA2 ChIP-seq data from the Gene Expression Omnibus (GEO). GEO IDs and additional information can be found in Supplemental Dataset 1. All datasets were analyzed using the same analysis pipeline, which is based on the ENCODE consortium standards and is described below.

ChIP-seq for transcription factors (BATF, Santa Cruz sc-100974; EBF1, Santa Cruz sc-137065; EBNA2, [PE2] Abcam ab90543; JUNB, Active Motif 39549; RBPJ, CST 5313; and SPI1, CST 2266) was performed using antibodies against each, in duplicate per cell line, using standard experimental procedures as described in Hong et al. 2021 [9]. Previous studies [40, 92] and our own data show that the EBNA2 PE2 antibody can recognize both EBNA2 type 1 and EBNA2 type 2 (Supplemental Figure 8). Cells were incubated in a crosslinking solution (1% formaldehyde, 5 mM 4-(2-hydroxyethyl)-1-piperazineëthanesulfonic acid (HEPES) pH 8.0, 10 mM sodium chloride, 0.1 mM ethylenediaminetetraacetic acid (EDTA), and 0.05 mM ethylene glycol tetraacetic acid (EGTA)) in Roswell Park Memorial Institute (RPMI) culture medium with 10% fetal bovine serum (FBS) and placed on a tube rotator at room temperature for 10 min. To stop crosslinking, glycine was added to a final concentration of 0.125 M, and the tubes were rotated at room temperature for 5 min. Cells were washed twice with ice-cold phosphate-buffered saline (PBS), resuspended in lysis buffer 1 (50 mM HEPES pH 8.0, 140 mM NaCl, 1 mM EDTA, 10% glycerol, 0.25% Triton X-100, and 0.5% NP-40), and incubated for 10 min on ice. Nuclei were harvested after centrifugation at 5,000 rpm for 10 min, resuspended in lysis buffer 2 (10 mM Tris–HCl pH 8.0, 1 mM EDTA, 200 mM NaCl, and 0.5 mM EGTA), and incubated at room temperature for 10 min. Protease and phosphatase inhibitors (Halt™ Protease and Phosphatase Inhibitor Cocktail (100X), Thermo Fisher Scientific, Waltham, MA) were included in both lysis buffers. Nuclei were resuspended in sonication buffer (10 mM Tris [pH 8.0], 1 mM EDTA, and 0.1% sodium dodecyl sulfate (SDS)). An S220 focused ultrasonicator (COVARIS, Woburn, MA) was used to shear chromatin (150–500-bp fragments) with 10% duty cycle, 175 peak power, and 200 bursts per cycle for 7 min. A portion of the sonicated chromatin was run on an agarose gel to verify fragment sizes. Sheared chromatin was pre-cleared with 10 μL of Protein A or G Dynabeads (Thermo Fisher Scientific) at 4 °C for 1 h.

Immunoprecipitation of TF-chromatin complexes was performed with an SX-8X IP-STAR compact automated system (Diagenode). Beads conjugated to antibodies against each TF were incubated with precleared chromatin at 4 °C for 8 h. The beads were then washed sequentially with wash buffer 1 (10 mM Tris–HCl [pH 7.5], 150 mM NaCl, 1 mM EDTA, 0.1% SDS, 0.1% NaDOC, and 1% Triton X-100), wash buffer 2 (10 mM Tris–HCl [pH 7.6], 400 mM NaCl, 1 mM EDTA, 0.1% SDS, 0.1% NaDOC, and 1% Triton X-100), wash buffer 3 (10 mM Tris–HCl [pH 8.0], 250 mM LiCl, 1 mM EDTA, 0.5% NaDOC, and 0.5% NP-40), and wash buffer 4 (10 mM Tris–HCl [pH 8.0], 1 mM EDTA, and 0.2% Triton X-100). Finally, the beads were resuspended in 10 mM Tris–HCl (pH 7.5) and used to prepare libraries via ChIPmentation [93].

The ChIP-seq libraries were sequenced as single-end 100-base reads on an Illumina NovaSeq 6000 at the Cincinnati Children’s Hospital Medical Center (CCHMC) Genomics Sequencing Facility, Cincinnati, Ohio. The ChIP-seq transcription factor Pipeline v2.0 from the ENCODE Project [94–96] (https://www.encodeproject.org/pipelines/) was used to perform QC assessments, genomic alignments, and peak calling. In brief, ChIP-seq reads were aligned to the human genome (hg19) using Bowtie2 (v. 2.3.4.3) [97]. Adaptors were trimmed using trimmomatic [98] (v. 0.39). Aligned reads were then sorted using samtools (v.1.9) [97] and duplicate reads were removed using Picard (v. 2.20.7) (https://broadinstitute.github.io/picard/). Peaks were called using the pipeline’s default parameters with MACS2 (v. 2.2.4) [99]. ENCODE “blacklist regions” were removed from the final peak set. Irreproducibility Discovery Rate (IDR) optimal peaks, generated from the ENCODE pipeline, were obtained and used for all downstream analyses. All public ChIP-seq datasets (Supplemental Dataset 1) were also analyzed using the same pipeline to obtain IDR peaks. To assess data quality, we created a rating system that measures “Data Quality” and “Library Complexity” based on ChIP-seq Encode Standards. In brief, “Library Complexity” was rated on a three-point scale. Library Complexity (based on NRF >  = 0.5; PBC1 >  = 0.5; PBC2 >  = 1) and Data Quality (10 million usable fragments per replicate; Self-consistency Ratio < 2 OR Rescue Ratio < 2; FRIP >  = 0.01) were graded on a 3 point scale. If the sample met all three requirements, it was marked as "Excellent", 2/3 requirements was categorized as "Good", and 1/3 requirements was categorized as “Fair”. “Altogether, 15 of the 19 ChIP-seq datasets were deemed ‘Excellent’, four were deemed ‘Good’, and none were deemed ‘Fair’ in terms of “Data Quality” using this system. With respect to “Library Complexity”, 13 of the 19 datasets were deemed ‘Excellent’ quality, three were deemed ‘Good’, and three were deemed ‘Fair’. Datasets that fell below the quality standards were not considered. Detailed QC metrics are provided in Supplemental Datasets 2, 3, and 4. Full QC information is provided in “Additional File 19 QC Reports.zip.”

### Assay for Transposase-Accessible Chromatin with sequencing (ATAC-seq)

Assay for Transposase-Accessible Chromatin with sequencing (ATAC-seq) is a method for determining chromatin accessibility across the genome. ATAC-seq data were generated and analyzed for the AG876, Jiyoye, and GM12878 and Mutu-III cell lines. Transposase Tn5 with sequencing adapter sequences was used to cut the accessible DNA, as detailed in Buenrostro et al. 2015 [100]. The resulting accessible DNA sequences were isolated, and libraries were prepared from 50,000 cells from each cell line using the OMNI ATAC protocol [101]. The libraries were sequenced on the Illumina NovaSeq 6000 (CCHMC Genomics Sequencing Facility) with 2 × 150 bp paired-end reads. The ENCODE ATAC-seq pipeline (v2.0) was used to perform QC assessments, genomic alignments, and peak calling [94–96] (https://www.encodeproject.org/pipelines/). In brief, ATAC-seq reads were aligned to the human genome (hg19) using Bowtie2 (v. 2.3.4.3) [97]. Adaptors were trimmed using trimmomatic [98] (v. 0.39). Aligned reads were then sorted using samtools (v.1.9) [102] and duplicate reads were removed using Picard (v. 2.20.7) (https://broadinstitute.github.io/picard/). Peaks were called using the pipeline’s default parameters with MACS2 (v. 2.2.4) [99]. To assess data quality, we created a rating system that measures “Data Quality” and “Library Complexity” based on ATAC-seq Encode Standards. In brief, “Library Complexity” was rated on a three-point scale (based on NRF >  = 0.7; PBC1 >  = 0.7; PBC2 >  = 1). If the sample met all three requirements, it was marked as "Excellent", 2/3 requirements was categorized as "Good", and 1/3 requirements was categorized as “Fair”. ATAC Data Quality (50 million usable fragments per replicate; Self-consistency Ratio < 2 OR Rescue Ratio < 2; Alignment rate > 80%; TSS > 6; FRIP > 0.3), was graded on a 5-point scale. If the sample met 4 or more requirements, it was marked as “Excellent”, 3/5 requirements was marked as "Good", and 2/5 requirements was marked as “Fair”. Altogether, two of the four ATAC-seq datasets were deemed ‘Excellent’, two were deemed ‘Good’, and none were deemed ‘Fair’ in terms of “Data Quality” using this system. With respect to “Library Complexity”, two of the four datasets were deemed ‘Excellent’ quality, two were deemed ‘Good’, and none were deemed ‘Fair’. Datasets that fell below the quality standards were not considered. Detailed QC metrics are provided in Supplemental Datasets 2, 3, and 4. Full QC information is provided in “Additional File 19 QC Reports.zip.”

### Identification of EBV type-specific genomic features

To identify type-specific genomic features (ChIP-seq and ATAC-seq peaks; see schematic in Fig. 2A), all events (peaks) that were found in either EBV-1 (GM12878 and Mutu-III) or EBV-2 (AG876 and Jiyoye) were first combined into the union of EBNA2-1 peaks and the union of EBNA2-2 peaks. Using bedtools v2.29.2 (https://bedtools.readthedocs.io/en/latest/index.html), we merged overlapping events to prevent duplicated peaks in downstream analysis. We next determined which events were found exclusively in EBV-1 or EBV-2 (bedtools subtract -A). This established EBV-1 or EBV-2 specific events, which were defined as events that do not overlap with the other EBV type (using bedtools default of 1E-9 or 1 bp). Shared peaks were determined by removing all type-specific peaks calculated previously and defined as any two peaks between EBV-1 and EBV-2 that overlap by at least 1 bp. Likewise, to obtain a dataset of all RBPJ events (Fig. 3C), we combined and merged all RBPJ peaks from the RBPJ datasets used in this paper (Supplemental Dataset 1), as detailed above. This was repeated for the other hTFs.

To identify stringent type-specific EBNA2 ChIP-seq peaks, we utilized bedtools v2.29.2 (https://bedtools.readthedocs.io/en/latest/index.html). For example, stringent EBNA2 type 1 specific peaks were determined by taking the intersect (bedtools intersect, default parameters) of Mutu-III and GM12878 (type 1) and subtracting the union of Jiyoye and AG876 (type 2, see above). The same approach was used to create stringent EBNA2 type 2 specific peaks. To create the “Shared Stringent” peaks, we first calculated the intersection (bedtools intersect, default parameters) of Mutu-III and GM12878 EBNA2 ChIP-seq peaks creating “All Type 1 EBNA2 Stringent Peaks”. An analogous approach was used to create “All Type 2 EBNA2 Stringent Peaks”. "Shared EBNA2 Stringent Peaks" was calculated by taking the intersection (bedtools intersect, default parameters) of “All Type 1 EBNA2 Stringent Peaks” and “All Type 2 EBNA2 Stringent Peaks."

To visualize differences in ChIP-seq or ATAC-seq signal strength within the type-specific regions (as depicted in Fig. 2C, and Supplemental Figures 2 and 3), Compute Matrix v3.5.1 and plotHeatmap v3.5.1 from deepTools (https://deeptools.readthedocs.io/en/develop/index.html) were used. For each cell line, the read enrichment within each peak contained in a ChIP-seq or ATAC-seq dataset (obtained from fc.bigwig files) was determined ± 2.5 kb from the peak center. This value was then visualized using the plotHeatmap function.

### Differential peak analysis using DiffBind

To determine statistically significant differential peaks between EBV-1 and EBV-2 datasets (ATAC-seq and ChIP-seq), DiffBind v3.10.1 [103] was used with default parameters (https://bioconductor.org/packages/release/bioc/html/DiffBind.html). Samples were categorized by EBV type (EBV-1 or EBV-2). Using the native normalization strategy and background binding estimation, differential peaks were identified. The alpha threshold was set to 0.05 with a Linear Fold Change Cutoff of 1.5.

### Gene set enrichment analysis using GREAT

GREAT [37] was used to perform gene set enrichment for genes near EBNA2 binding sites for the gene ontology (GO) biological process category [104]. We ran GREAT with its default settings, where each gene is assigned a regulatory domain (for proximal: 5 kb upstream, 1 kb downstream of the TSS; for distal: up to 1 Mb). We defined significant results as those with an FDR-corrected one-tailed binomial test *p*-value < 0.05. To determine which GO terms were impacted by EBNA2 type, we defined unequal enrichment when the difference in the normalized FDR between type-specific peaks was greater than 20%.

### Transcription Factor (TF) DNA binding motif enrichment analysis

Human TF motif enrichment analysis was performed using the Hypergeometric Optimization of Motif EnRichment (HOMER) software package [105]. A modified version of HOMER was used, which incorporates human motifs obtained from Cis-BP build 2.0 [106] and uses a log base 2 likelihood scoring system. We used representative hTF motifs (AP-1 [BATF], M09495_2.00; EBF1, M09527_2.00; RBPJ, M10413_2.00; and SPI1, M04805_2.00) for further analyses (Fig. 3B). These example motifs were chosen as representatives because they had high motif enrichment in EBNA2 type 1 and/or EBNA2 type 2 specific peaks (Supplemental Dataset 3) and are strong matches to the established motif for their respective hTFs. HOMER was also used to produce QC metrics to determine if newly generated ChIP-seq and ATAC-seq peak datasets were enriched for expected hTF motifs (e.g., the RBPJ ChIP-seq dataset should be highly enriched for RBPJ motifs). See Supplemental Dataset 3 for the full HOMER results of these analyses.

### Estimation of the significance of the intersection between a set of genomic coordinates and a library of TF ChIP-seq peaks using RELI

The Regulatory Element Locus Intersection (RELI) algorithm [5, 107, 108] was used to estimate the significance of overlap between genomic features generated in this study (e.g., EBNA2 and hTF ChIP-seq co-occupancy events, as depicted in Fig. 3C; TF binding events intersecting GWAS Disease SNPs, as depicted in Fig. 5). As input, RELI takes the genomic coordinates from a set of genomic features. RELI then systematically intersects these coordinates with each member of a large library of ChIP-seq datasets one at a time, and the number of input regions overlapping the peaks of each dataset is counted. Next, the significance of the intersection of each dataset is calculated (*p*-value) using a simulation-based procedure in which the peaks from the input dataset are randomly distributed within the union coordinates of open chromatin from human cells. A distribution of expected overlap values is then created from 2,000 iterations of random sampling from this negative set, each time choosing a set of negative examples that match the input set in terms of the total number of genomic loci. The distribution of the expected overlap values from the randomized data resembles a normal distribution and can thus be used to generate a Z-score and corresponding *p*-value estimating the significance of the observed number of input regions that overlap each dataset. This procedure is then completed for all datasets in the ChIP-seq library. *P*-values are corrected using Bonferroni’s method.

To determine the significance of the overlap between our EBNA2 ChIP-seq datasets and GWAS-derived disease-associated genetic variants, we generated a custom GWAS catalogue that combines all of the ancestries for a particular disease. To this end, we downloaded the Genome Wide Association Studies Catalogue (https://www.ebi.ac.uk/gwas/) v1.0.2, as queried on June 20th 2019. Independent risk loci for each disease/phenotype were identified based on linkage disequilibrium (LD) pruning (*r*2 < 0.2). Risk loci across these independent genetic risk variants were identified by linkage disequilibrium expansion (*r*2 > 0.8) based on 1000 Genomes Data using PLINK (v.1.90b). This created a list of disease risk loci, along with the corresponding genetic variants within the LD block. Finally, the LD expanded list for each ancestry was merged by disease, creating a single list of variants for a given disease. This list of variants was then used for RELI analyses.

RELI was also used as a QC metric to determine if newly generated ChIP-seq and ATAC-seq datasets overlap significantly with relevant public datasets (e.g., RBPJ ChIP-seq peaks should overlap significantly with published RBPJ ChIP-seq peaks). See Supplemental Dataset 4 for the full RELI results.

### Western blots

Western blots were used to assess protein levels in EBV-1 (GM12878 and Raji) and EBV-2 (Jiyoye and AG876) infected cells. To obtain nuclear lysates (three biological replicates) for Western blots, cells lines were resuspended in 1 mL of cold PBS per 10 million cells. Cells were then centrifuged at 4 °C, 300 × g for 5 min and the PBS was aspirated off. Cell pellets were resuspended in 400μl of CE buffer (10 mM HEPES, pH 8; 10 mM KCl; 0.1 mM EDTA; 1 mM DTT; 1 × Halt protease and phosphatase inhibitor) and incubated for 15 min on ice. Afterwards, 25μl of 10% Nonidet P-40 were mixed into the solution and the samples were centrifuged at max speed (17.3 × g) at 4 °C for 3 min. After discarding the supernatant, the cell pellet was resuspended in 30μl of NE Buffer (20 mM HEPES, pH 8; 0.4 M NaCl; 1 mM EDTA; 1 mM DTT; 1 × Halt phosphatase inhibitor). Cells were sonicated using the Q125 sonicator (Qsonica) at 20% power, 5 s pulses for a total of 15 s. The supernatant was stored at -70 °C. 5.5μl of aliquot was used to measure protein concentration using the bicinchoninic acid assay (BCA) (Thermo Fisher Scientific).

Nuclear lysates of samples were mixed with loading buffer and DTT and heated at 95° C for 2 min. After cooling, the samples were loaded into a 4–12% Nu page Bis–Tris Gel (Invitrogen) and ran for 90 min at 130 V in MOPS buffer, or 65 min at 130v in MES buffer. The gel was then transferred to a PVDF membrane using the iBlot Machine (Thermo Fisher Scientific). For Western blot normalization, membranes were stained using the Revert™ 700 Total Protein Stain (Licor Bioscience). The membrane was blocked in Intercept Blocking Buffer for 1 h at room temperature. The primary antibody was diluted (BATF, CST 8638, 1:1000; EBF1, Santa Cruz sc-137065, 1:500; EBNA2, [PE2] Abcam ab90543, 1:1000; JUNB, Active Motif 39,549, 1:1000; RBPJ, CST 5313, 1:1000; and SPI1, CST 2266, 1:1000) in Intercept Blocking Buffer (with Tween 20 diluted at 1:1000) and the membranes incubated in primary antibody overnight at 4 °C with rocking. Membranes were washed in a PBS/Tween 20 solution twice and were incubated in fluorescently labeled secondary antibody in Intercept Blocking Buffer (with Tween 20 and SDS) for 60 min at room temperature with rocking. Membranes were washed again in a PBS/Tween 20 solution 2 times before being imaged using the Odyssey DLx Imaging System (Licor Bioscience). Protein expression levels were determined using Empiria Studio v2.2 (Licor Bioscience).

### Co-immunoprecipitation (Co-IP) assays

Co-IP was used to pull down known EBNA2 partners in EBV-1 and EBV-2 infected cell lines. To obtain nuclear lysates, cell lines were resuspended in 1 mL of cold PBS per 10 million cells. Cells were then centrifuged at 4 °C, 300 × g for 5 min and the PBS was aspirated off. Cell pellets were resuspended in 400μl of CE buffer (10 mM HEPES, pH 7.0; 10 mM KCl; 0.1 mM EDTA; 1 mM DTT; 1 × Halt phosphatase inhibitor) and incubated for 15 min on ice. Afterwards, 25μl of 10% Nonidet P-40 were mixed into the solution and the samples were centrifuged at max speed (17.3 × g) at 4 °C for 3 min. The supernatant, which contains the cytoplasmic portion, was discarded. The remaining nuclei samples were washed twice in 500μl of cold PBS. Samples were spun for 1 min at max speed (17.3 × g). After discarding the PBS supernatant, the cell pellet was resuspended in 500μl of Co-IP Buffer (1% NP-40; 150 mM NaCl; 10 mM Tris–HCl, pH 7.4; 1 mM EDTA, pH 8.0; 3% Glycerol; H_2_O). Samples were left on ice for 10 min. Afterwards, cells were sonicated using the Q125 sonicator (Qsonica) at 20% power, 5 s pulses for a total of 15 s. Samples were spun down for 10 min at max speed and the nuclear lysate was collected. 5.5μl of aliquot was used to measure protein concentration using the bicinchoninic acid assay (BCA) (Thermo Fisher Scientific). 100ug of nuclear lysate were aliquoted for input with 2 × Laemmli loading buffer and stored at -80C.

To begin co-immunoprecipitation, 500ug lysates in Co-IP buffer were aliquoted to a final volume of 500uL. EBNA2 antibody (PE2 Abcam ab90543; 1:100) was added to the Co-IP sample and rotated at 4C for 1 h. Protein A/G beads were washed 2 times with Co-IP buffer and then added to Co-IP samples at 1:10 dilution and rotated for 1 h at 4 degrees. Tubes were placed on a magnetic rack and the clear liquid was collected and discarded. The remaining beads were washed with 500μl Co-IP buffer four times. After washing, 50μl heated sample buffer was added to the IP sample and mixed.

IP and input samples were heated at 70C for 5 min. After cooling, the samples were loaded into a 4–12% Nu page Bis–Tris Gel (Invitrogen) and ran for 90 min at 130v in MOPS buffer. The gel was then transferred to a PVDF membrane using the iBlot Machine (Thermo Fisher Scientific). Samples were blocked in 5% milk in PBST (50 ml PBST plus 2.5 g dehydrated milk powder) for at least 1 h. The primary antibody (EBF1, CST 50752; RBPJ, CST 5313; and SPI1, CST 2266) was diluted in 5% milk in PBST at a 1:1000 ratio and the membranes incubated in primary antibody overnight at 4 °C with rocking. Membranes were washed in a PBST solution 3 times for 5 min each and were incubated in HRP conjugated secondary antibody in 5% milk in PBST for 1 h at room temperature with gentle shaking. Membranes were washed again in a PBST solution 3 times for 5 min each. Then 2 ml of SuperSignal West Femto Maximum Sensitivity Substrate was added to each blot and incubated for 2 min. Substrate was removed before being imaged with the ChemiDoc Touch Imaging System (Bio-Rad).

### RNA-sequencing (RNA-seq)

RNA sequencing (RNA-seq) was used to measure human gene expression levels in EBV-1 (GM12878 & Mutu-III) and EBV-2 (AG876 & Jiyoye) infected cell lines. We generated new RNA-seq datasets for the AG876, Jiyoye, and GM12878 cell lines. Public Mutu-III RNA-seq datasets (see Supplemental Dataset 1) were also included. The mirVana RNA (Thermo Fisher Scientific) isolation kit was used to isolate total RNA from 3–5 million cells of each cell line in triplicates (at greater or equal to 90% viability) following the manufacturer’s recommended procedures. Libraries were prepared from RNA samples using the TruSeq Stranded Total RNA with Ribo-Zero Globin (Illumina, Inc., San Diego, CA) and sequenced at 150 paired end bases (50 M reads per sample) at the CCHMC Genomics Sequencing Facility.

Raw RNA-seq datasets were processed using the nf-core pipeline [109, 110] (v. 3.11.1) (https://nf-co.re/rnaseq). In brief, the samples underwent QC using FastQC v. 0.11.9 (https://www.bioinformatics.babraham.ac.uk/projects/fastqc/) and adaptors were trimmed and filtered using Cut Adapt v. 3.4 (https://cutadapt.readthedocs.io/en/stable/). Other tools, such as RseqQC [111, 112], Qualimap [113, 114], dupRadar [115], and Preseq (https://github.com/smithlabcode/preseq), were used to assess the quality of the datasets. Sequencing data were aligned to a modified hg19 genome containing transcript sequences for EBV-1 (ASM240226v1 / GCF_002402265.1) or EBV-2 (ViralProj20959 / GCF_000872045.1), respectively using STAR v. 2.7.9a (https://github.com/alexdobin/STAR) [116]. Ribosomal RNA was removed from the aligned datasets using the Silva Library (https://www.arb-silva.de/) and SortmeRNA v. 4.3.4 (https://bioinfo.lifl.fr/RNA/sortmerna/) to eliminate potential contamination from non-target RNA species. Transcript abundance estimates were calculated using the GENCODE (v. 19) annotation for hg19 with additional EBV transcripts (see above) added with Salmon (v.1.10.1) (https://combine-lab.github.io/salmon/). Normalized gene read counts were calculated using DESeq2 [117] utilizing an alpha threshold of 0.05 and a Linear Fold Change Cutoff of 2. All datasets, including public datasets, were analyzed using the same pipeline. All datasets passed standard QC procedures and showed strong agreement between replicates (Supplemental Dataset 2). Transcripts per million (TPM) were generated (Supplemental Dataset 6). Full QC information is provided in “Additional File 19 QC Reports.zip.”

### EBNA2/EBF1 split nanoluciferase assays

HEK-293 cells were plated on 6 well plates and transfected the following day using Lipofectamine 2000 (Thermo Fisher Scientific) following the manufacturer’s instructions. The control cells received 0.5 mg of a plasmid encoding GFP while the experimental wells received 0.5 mg of SmBit-EBF1 fusion plasmid and either 0.5 mg of B95.8 LgBit-EBNA2 (B95.8) or 0.125 mg of LgBit-EBNA2 (AG876) fusions to compensate for the lower expression of the B95.8 EBNA2. We selected the amount of DNA to transfect such that the amount of protein was relatively the same. Because B95.8 EBNA has a stretch of prolines [118–120], the translation of type 1 B95.8 is less efficient. Thus, twice as much B95.8 EBNA2 was transfected relative to AG876 EBNA2. The following day the transfected cells were counted, and 100,000 cells of each condition were plated in triplicate on a 96 well plate and the remaining cells were replated on 6 well plate to collect parallel samples for Western blot (see above). The next day the cells on the 6 well plate were lysed in RIPA-Doc plus protease inhibitors, quantified using the BCA protein assay kit and equivalent amount of protein lysates were used for Western blot for the SmBit-EBF1 and LgBit-EBNA2 fusions. For the nanoluciferase assay, the media on the cells on the 96 well plate was changed to 50 ml of Opti-MEM (Thermo Fisher Scientific) plus 12.5 ml of Promega Nano-Glo Live Cell Detection (Promega) reagent and luminescence was read on a GloMax (Promega) plate reader with a 10 s integration time.

### EBF1 ChIP-qPCR

Equivalent amounts of EBF1 ChIP libraries from GM12878 or AG876 cells were diluted 0.01 ng/μl. The amount of specific genomic locations present in the diluted ChIP libraries were measured in triplicate by qPCR. Briefly, 5 μl of the diluted ChIP libraries were added to a mastermix of the target site primers (0.75 μl each of primers resuspended at 10 mM), 6 μl water, and 12.5 μl of 2X SYBR Green (Thermo Fisher Scientific)) on a 96 well qPCR plate. The qPCR was then run and read on an Applied Biosystems QuantStudio Real-Time PCR machine. The GM12878 and AG876 signal for each genomic location was normalized using the CT of the AG876 samples to determine the relative enrichment of EBF1 ChIP from GM12878 compared to AG876.

### Supplementary Information

**Supplementary Material 1.**

**Supplementary Material 2.**

**Supplementary Material 3.**

**Supplementary Material 4.**

**Supplementary Material 5.**

**Supplementary Material 6.**

**Supplementary Material 7.**

**Supplementary Material 8.**

**Supplementary Material 9.**

**Supplementary Material 10.**

**Supplementary Material 11.**

**Supplementary Material 12.**

**Supplementary Material 13.**

**Supplementary Material 14.**

**Supplementary Material 15.**

**Supplementary Material 16.**

**Supplementary Material 17.**

**Supplementary Material 18.**

**Supplementary Material 19.**

## Data Availability

All data, including. fastq files, .bam files, .bw files, called peaks, type-specific peak locations, and TPM values (for RNA-seq) are available in the Gene Expression Omnibus (GEO) database under accession number GSE246062 All functional genomics data analyzed in this study are available as a UCSC Genome Browser Track Hub: https://genome.ucsc.edu/s/Ledsall/EBNA2_Type_2.

## Abbreviations

Co-IP: Co-immunoprecipitation
EBV: Epstein-Barr Virus
EBNA2: Epstein-Barr Nuclear Antigen 2
ESE: EBNA2 Super Enhancers
FriP: Fraction of Reads in Peaks
GWAS: Genome Wide Association Study
hTF: Human transcription factor
IBD: Inflammatory bowel disease
JIA: Juvenile idiopathic arthritis
LCL: Lymphoblastoid cell line
MS: Multiple sclerosis
PBC: Primary biliary cholangitis
QC: Quality Control
RA: Rheumatoid arthritis
SLE: Systemic lupus erythematosus
T1D: Type 1 diabetes
TPM: Transcripts per million
TSS: Transcription start site
UC: Ulcerative colitis
