# Supplementary material for: Shared and distinct interactions of type 1 and type 2 Epstein-Barr Nuclear Antigen 2 with the human genome
Source: BMC Genomics. 2024 Mar 12;25:273. doi: 10.1186/s12864-024-10183-8 (PMC10935964; doi:10.1186/s12864-024-10183-8)
Supplement: Supplementary file 1 — Supplementary Material 1. [file 12864_2024_10183_MOESM1_ESM.pdf]

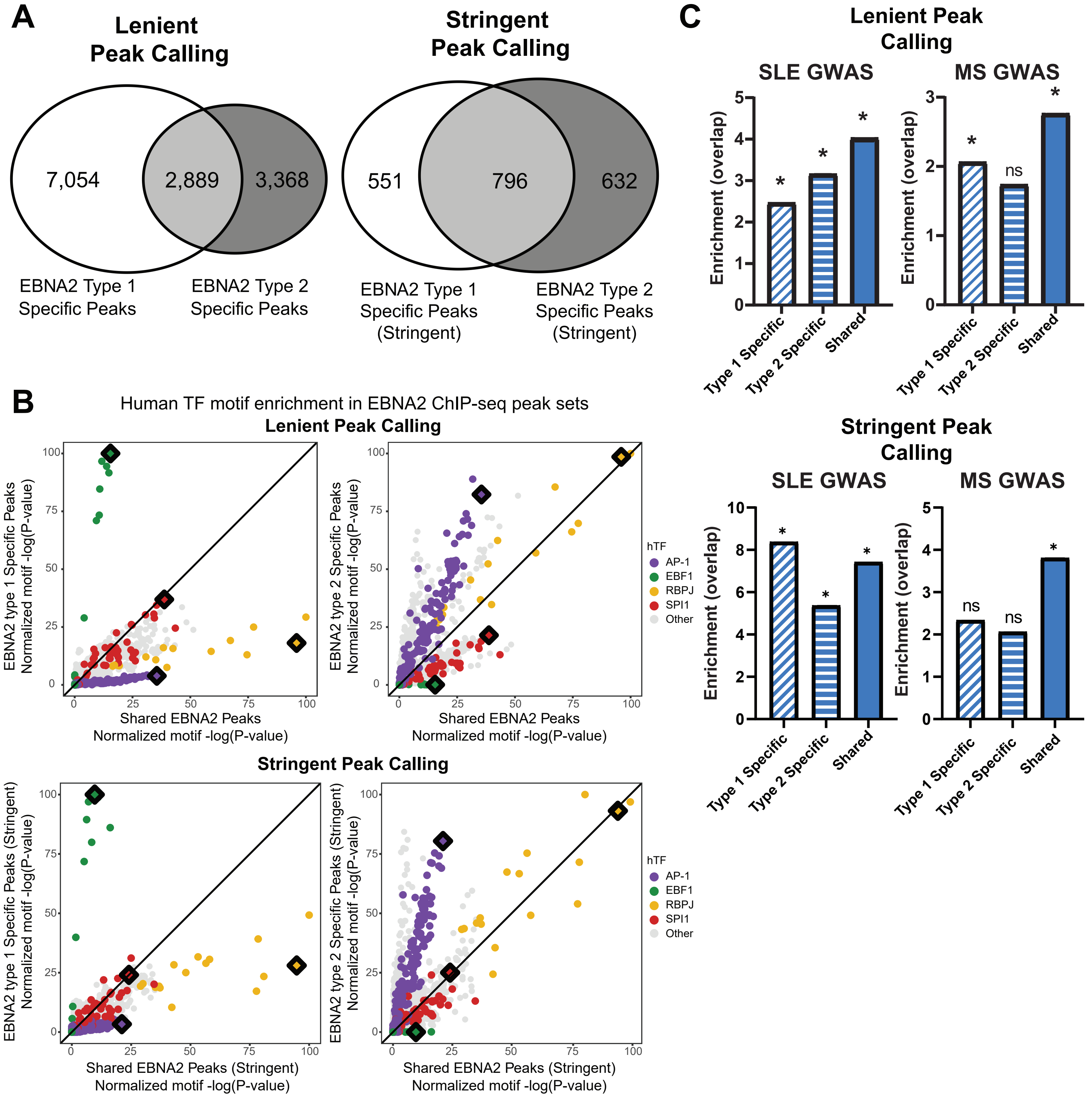

Additional File 1 Supplemental Figure 1: Analysis of stringent EBNA2 type-specific peak sets. Comparison of results between "lenient" and "stringent" EBNA2 type-specific peak sets (see Methods). A) Shared and type-specific EBNA2 peak counts. B) Unbiased computational prediction of EBNA2 human cofactors. Human transcription factor (hTF) motif enrichment analysis was performed within EBNA2 type 1 specific, type 2 specific, and shared peaks. Each dot represents the normalized significance of one hTF motif. Type 1 specific (y-axis, left) or type 2 specific (y-axis, right) normalized motif significance is compared to shared peaks (x-axis in both panels). Black diamonds indicate exemplar hTF motifs for the four TF classes that are depicted in Figure 3B. The black line indicates equivalent significance between the compared peak sets. Motifs are colored by class. C) Enrichment of type-specific and shared EBNA2 peak sets at disease risk loci for diseases previously established for type 1 EBNA2 (multiple sclerosis (MS) and systemic lupus erythematosus (SLE)). Datasets with significant overlap (as calculated by RELI) are marked with asterisks ($P<0.05$).
