# Supplementary material for: Shared and distinct interactions of type 1 and type 2 Epstein-Barr Nuclear Antigen 2 with the human genome
Source: BMC Genomics. 2024 Mar 12;25:273. doi: 10.1186/s12864-024-10183-8 (PMC10935964; doi:10.1186/s12864-024-10183-8)
Supplement: Supplementary file 2 — Supplementary Material 2. [file 12864_2024_10183_MOESM2_ESM.pdf]

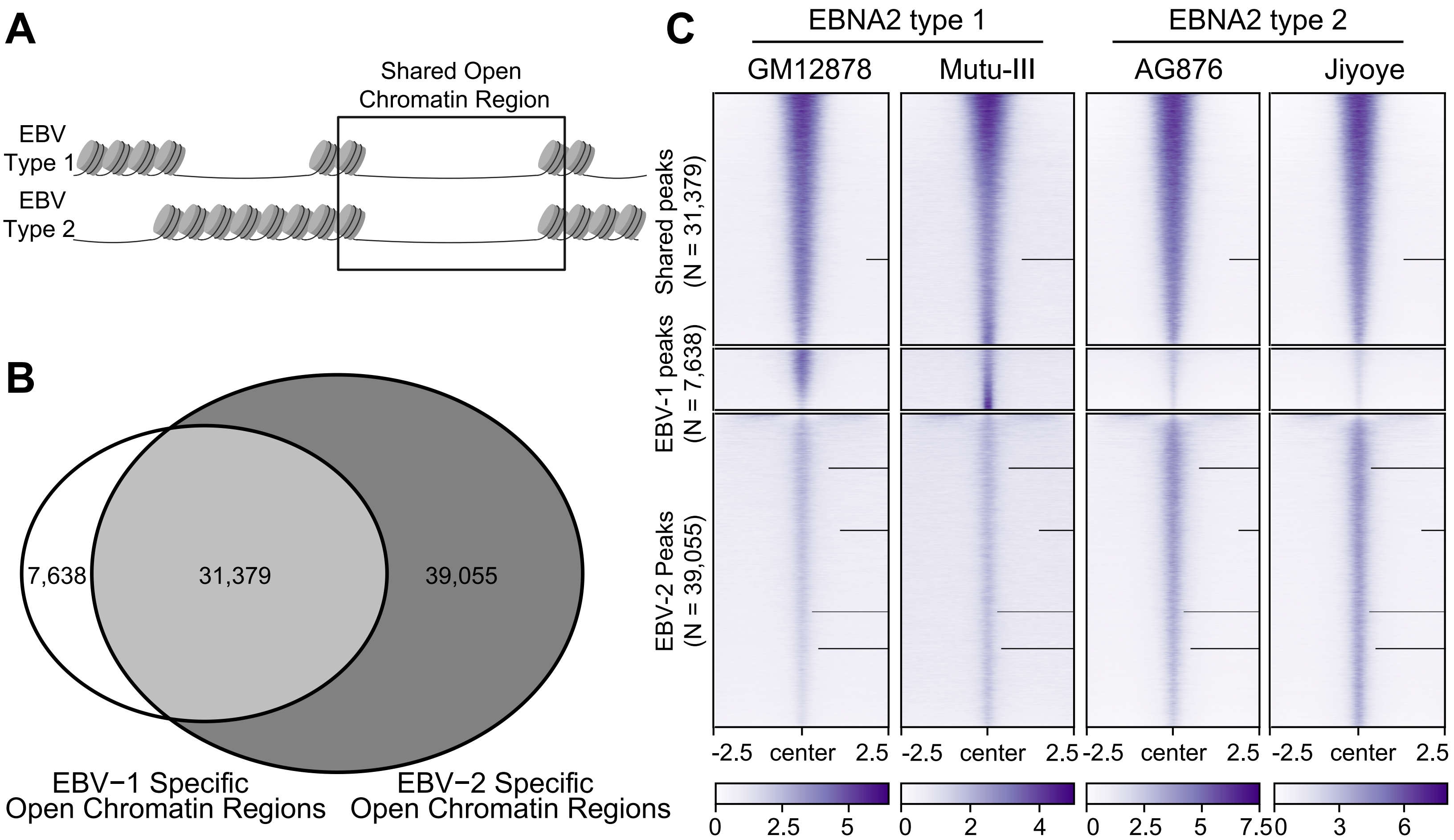

Additional File 2: Supplemental Figure 2. Shared and EBV type-dependent chromatin accessibility across the human genome. A) Schematic for identification of shared open chromatin regions (ATAC-seq peaks). B) Numbers of shared and type-specific open chromatin regions. C) Signal strength (normalized read depth) of EBV-1 (GM12878, Mutu-III) and EBV-2 (AG876, Jiyoye) ATAC-seq at shared and type-specific regions. As expected, shared peaks (top) have equal signal strength between the four cell lines. EBNA2 type 1 specific peaks have greater signal strength in EBV-1 cell lines (middle left) compared to EBV-2 cell lines (middle right). Likewise for type 2 (bottom). See Methods for ATAC-seq analysis details.
