# Supplementary material for: Shared and distinct interactions of type 1 and type 2 Epstein-Barr Nuclear Antigen 2 with the human genome
Source: BMC Genomics. 2024 Mar 12;25:273. doi: 10.1186/s12864-024-10183-8 (PMC10935964; doi:10.1186/s12864-024-10183-8)
Supplement: Supplementary file 3 — Supplementary Material 3. [file 12864_2024_10183_MOESM3_ESM.pdf]

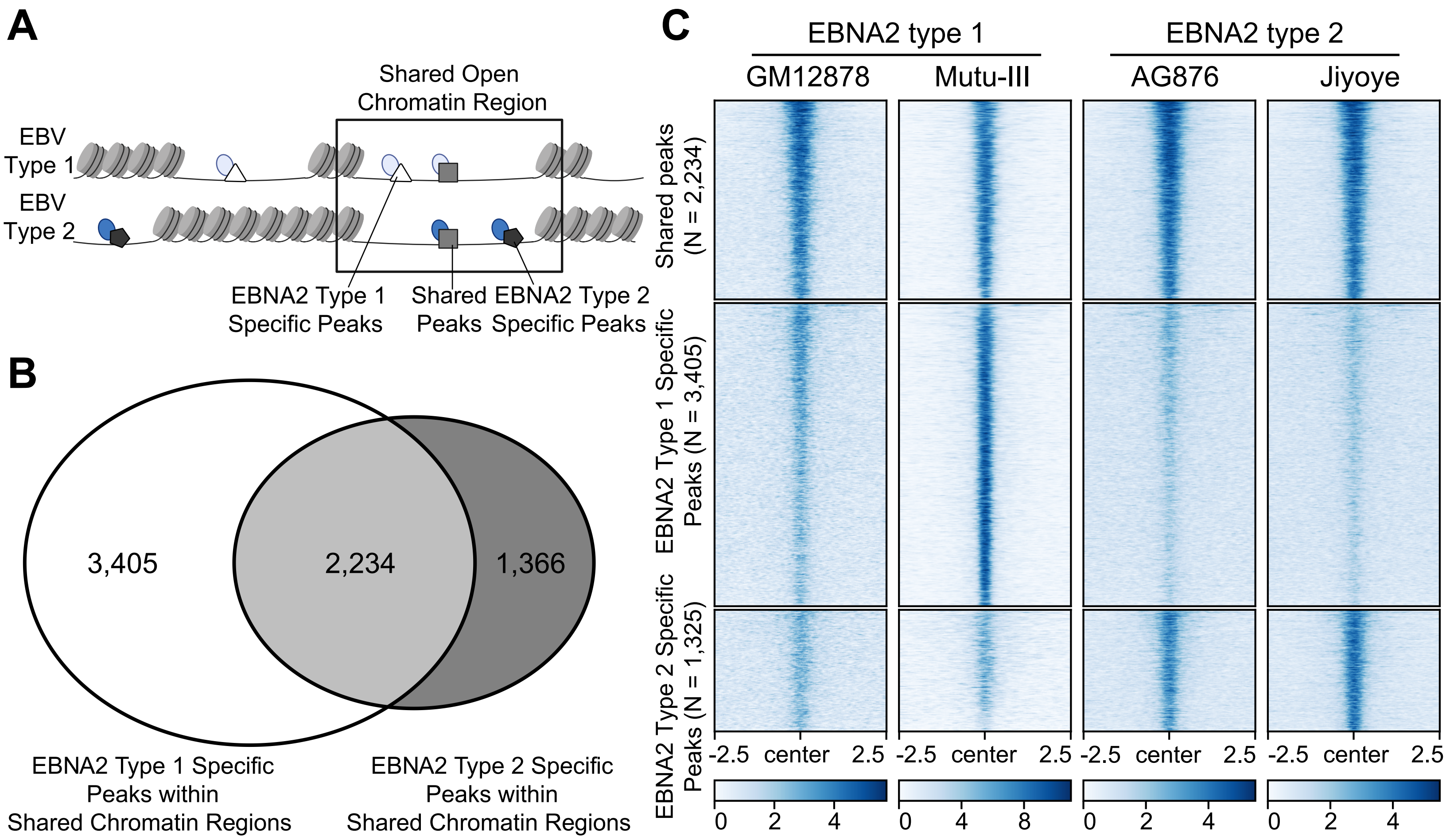

Additional File 3: Supplemental Figure 3. Shared and EBNA2 type-specific interactions within shared accessible loci across the human genome. A) Schematic of identification of type 1 and 2 EBNA2 ChIP-seq peaks within shared open chromatin regions. B) Shared and type-specific EBNA2 peak counts. C) ChIP-seq signal strength (normalized read depth) for type 1 (GM12878, Mutu-III) and type 2 (AG876, Jiyoye) EBNA2 at shared and type-specific regions located within regions of shared open chromatin. As expected, shared peaks (top) have equal signal strength between the four cell lines. EBNA2 type 1 specific peaks have greater signal strength in EBV-1 cell lines (middle left) compared to EBV-2 cell lines (middle right). Likewise for type 2 (bottom). See Methods for EBNA2 ChIP-seq analysis details.
