# Supplementary material for: Shared and distinct interactions of type 1 and type 2 Epstein-Barr Nuclear Antigen 2 with the human genome
Source: BMC Genomics. 2024 Mar 12;25:273. doi: 10.1186/s12864-024-10183-8 (PMC10935964; doi:10.1186/s12864-024-10183-8)
Supplement: Supplementary file 4 — Supplementary Material 4. [file 12864_2024_10183_MOESM4_ESM.pdf]

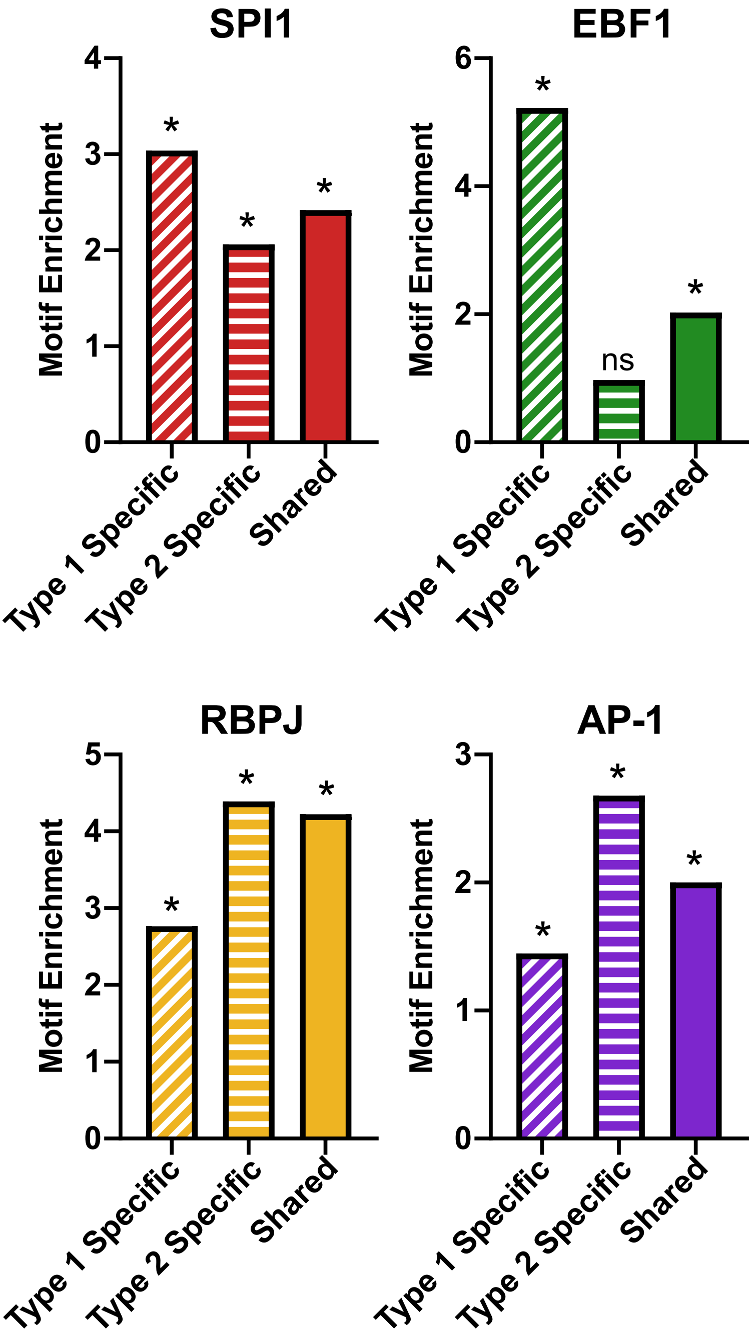

Additional File 4: Supplemental Figure 4. Human transcription factor motif enrichment for known and potential EBNA2 cofactors. Enrichment occurrence of exemplary hTF motifs (Figure 3, black diamonds) in EBNA2 shared and type-specific peak sets. For the four exemplary motifs, the enrichment (%foreground divided by %background) of predicted binding sites for the motif is shown. Asterisks indicate significant motif enrichment ($P<0.05$), as calculated by HOMER.
