# Supplementary material for: Shared and distinct interactions of type 1 and type 2 Epstein-Barr Nuclear Antigen 2 with the human genome
Source: BMC Genomics. 2024 Mar 12;25:273. doi: 10.1186/s12864-024-10183-8 (PMC10935964; doi:10.1186/s12864-024-10183-8)
Supplement: Supplementary file 5 — Supplementary Material 5. [file 12864_2024_10183_MOESM5_ESM.pdf]

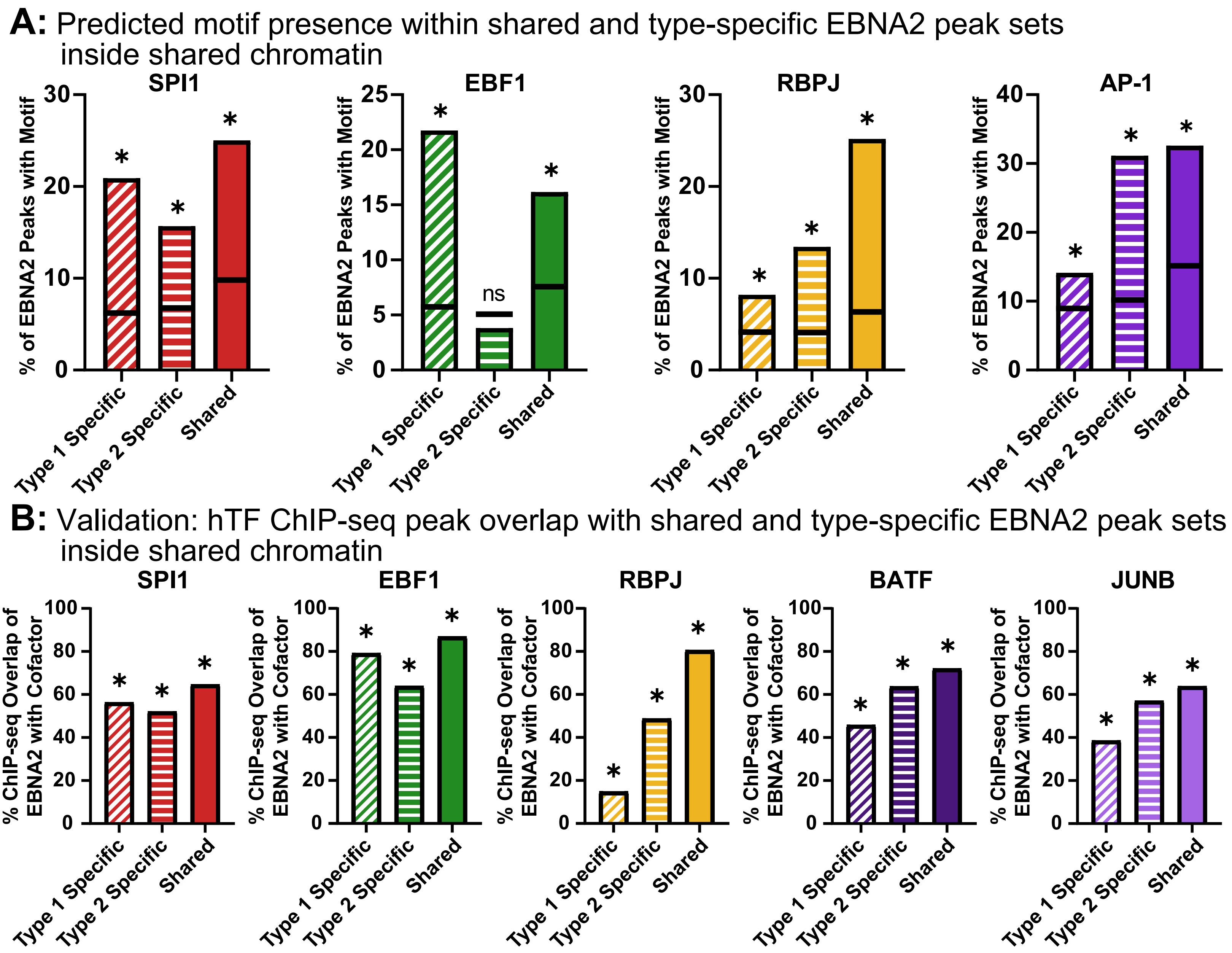

Additional File 5: Supplemental Figure 5. Identification of shared and type-specific EBNA2 hTF partners within shared accessible loci across the human genome. A) Frequency of occurrence of exemplary motifs in EBNA2 shared and type-specific peak sets located within shared accessibility loci. For the four exemplary motifs, the percent of peaks containing predicted binding sites for the motif is shown. Each bar represents percent foreground (i.e., the percent of actual peak DNA sequences containing a match to the motif). The horizonal black line within each bar depicts percent background (i.e., the percent of randomly selected genome sequences, matching GC content). Asterisks indicate significant motif enrichment (P<0.05), as calculated by HOMER. B) Experimental validation of predicted EBNA2 co-occupancy with hTFs inside regions of shared chromatin. Co-occupancy was assessed by hTF and EBNA2 ChIP-seq peak overlap. The hTFs BATF and JUNB were chosen as representative AP-1 family members (see Results). For each of the five hTFs, a union peak set was created by combining peaks across all cell lines. For each bar, the percent of each hTF union peak set overlapping each EBNA2 ChIP-seq peak category is shown. Datasets with significant overlap between EBNA2 peak sets and the union peak set of hTFs (as calculated by RELI) are indicated with asterisks (P<0.05). Note the consistency between the motif-based predictions (A) and ChIP-seq experimental validation results (B).
