# Supplementary material for: Shared and distinct interactions of type 1 and type 2 Epstein-Barr Nuclear Antigen 2 with the human genome
Source: BMC Genomics. 2024 Mar 12;25:273. doi: 10.1186/s12864-024-10183-8 (PMC10935964; doi:10.1186/s12864-024-10183-8)
Supplement: Supplementary file 6 — Supplementary Material 6. [file 12864_2024_10183_MOESM6_ESM.zip › Additional File 6_with_raw_images.pdf]

Additional Figure 6

Raw Images:

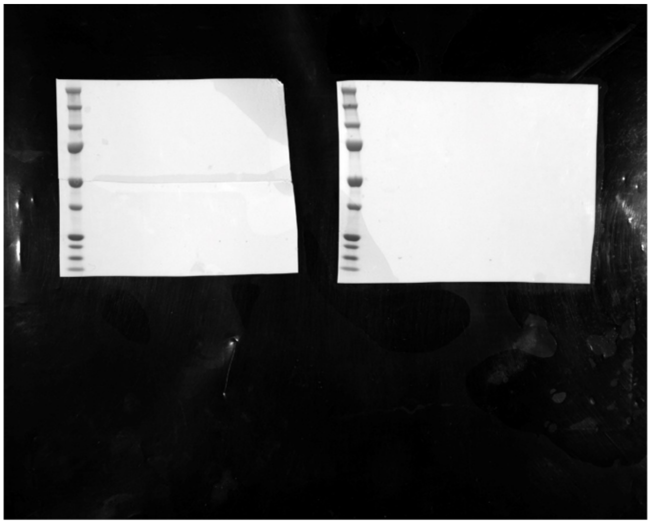

Light Image

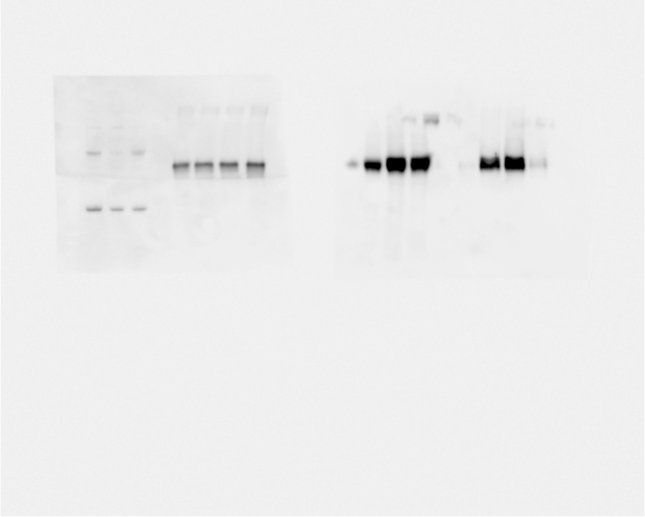

Low exposure time

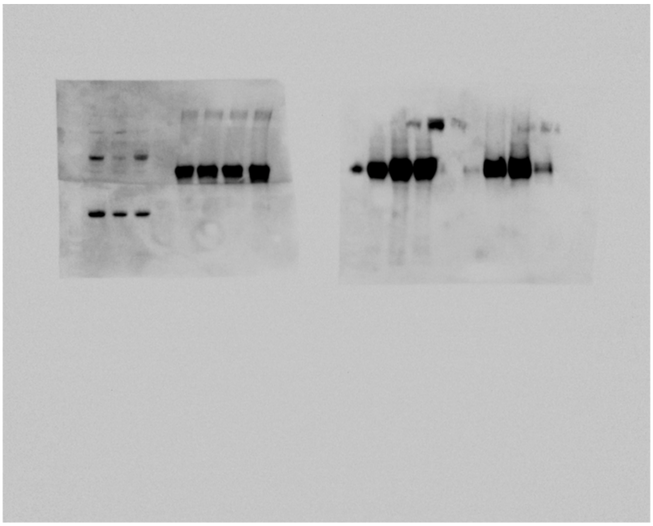

Low exposure time

Labelled Raw Images:

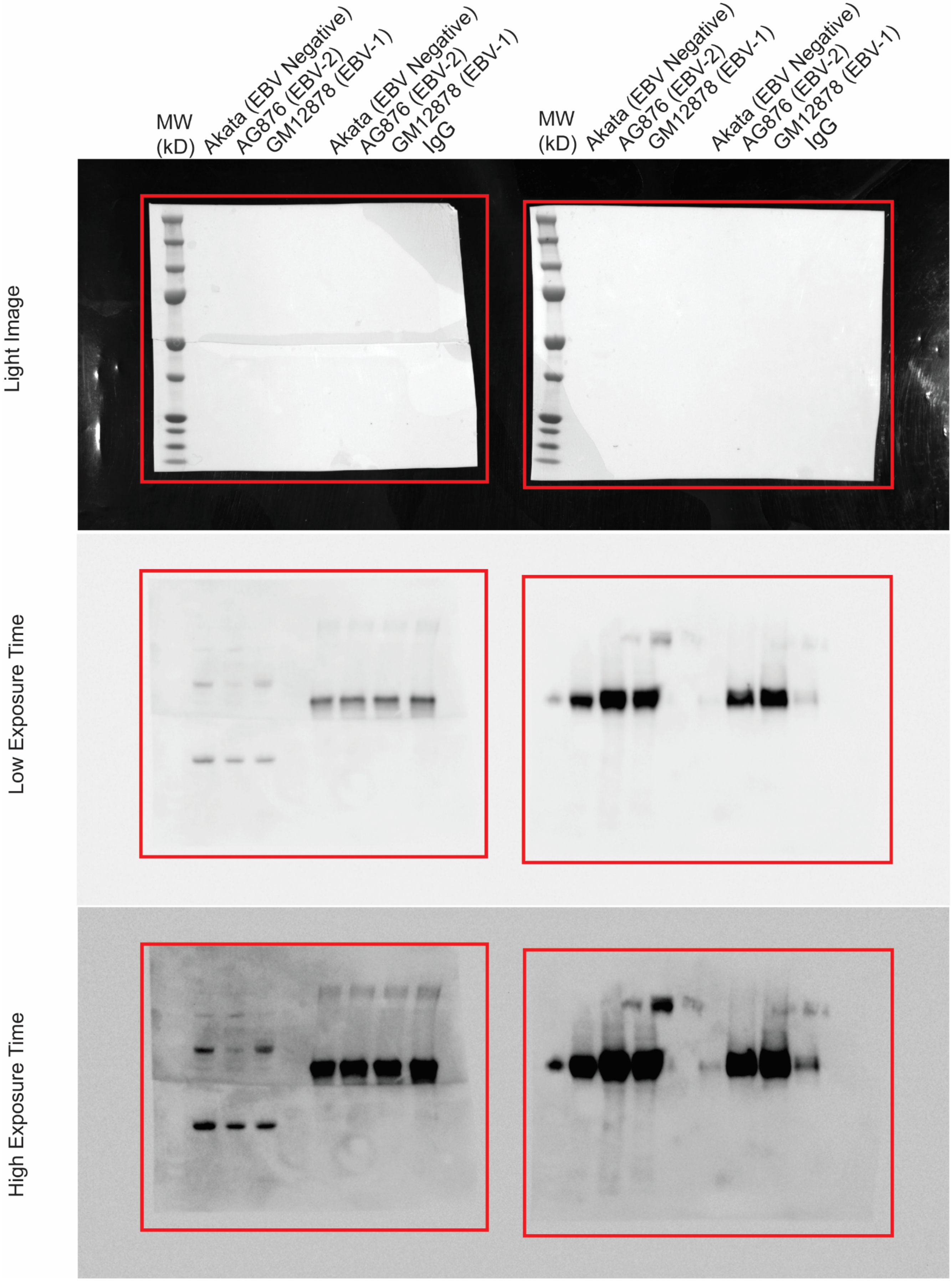

Final Image for Paper:

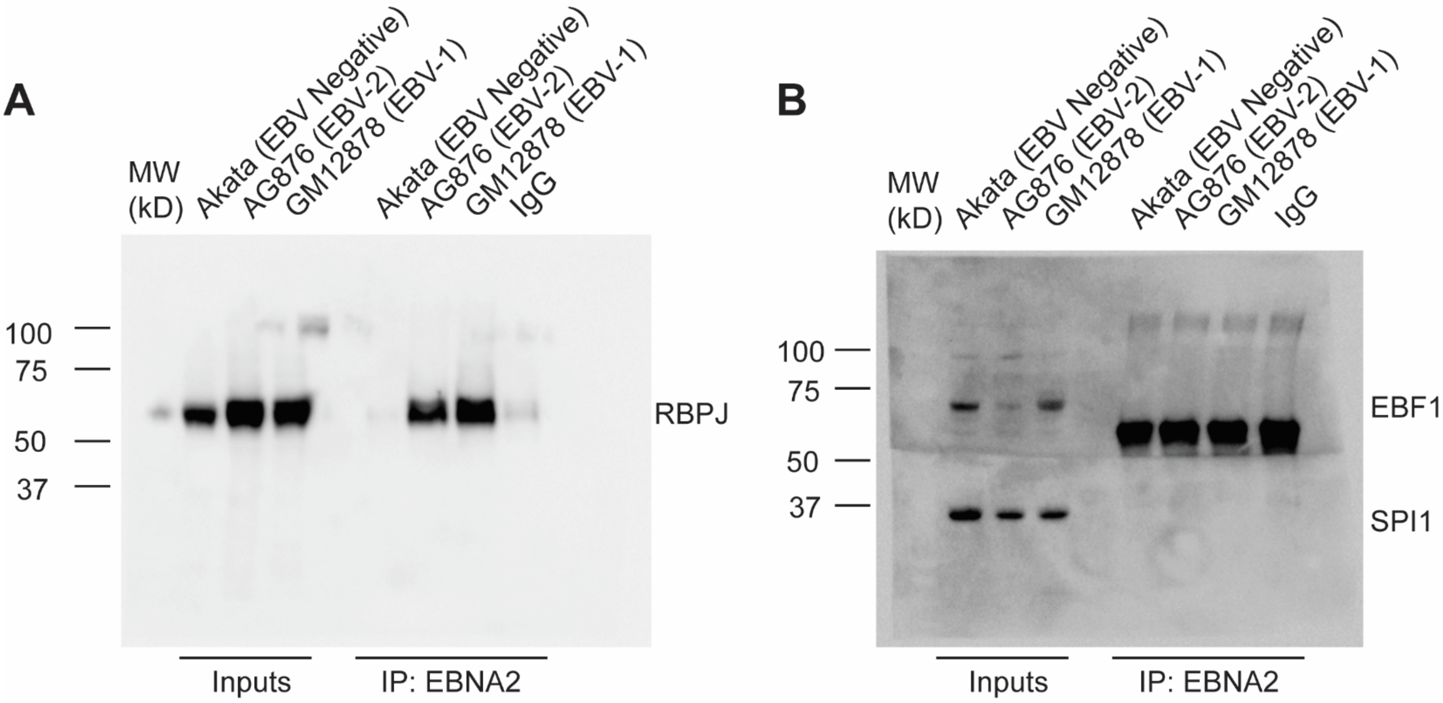

Note that panel A is in the blot on the right and panel B is the blot on the left of the raw images.
