# Supplementary material for: Shared and distinct interactions of type 1 and type 2 Epstein-Barr Nuclear Antigen 2 with the human genome
Source: BMC Genomics. 2024 Mar 12;25:273. doi: 10.1186/s12864-024-10183-8 (PMC10935964; doi:10.1186/s12864-024-10183-8)
Supplement: Supplementary file 6 — Supplementary Material 6. [file 12864_2024_10183_MOESM6_ESM.zip › Additional_File_6_Supplemental_Figure_6_REVISED.pdf]

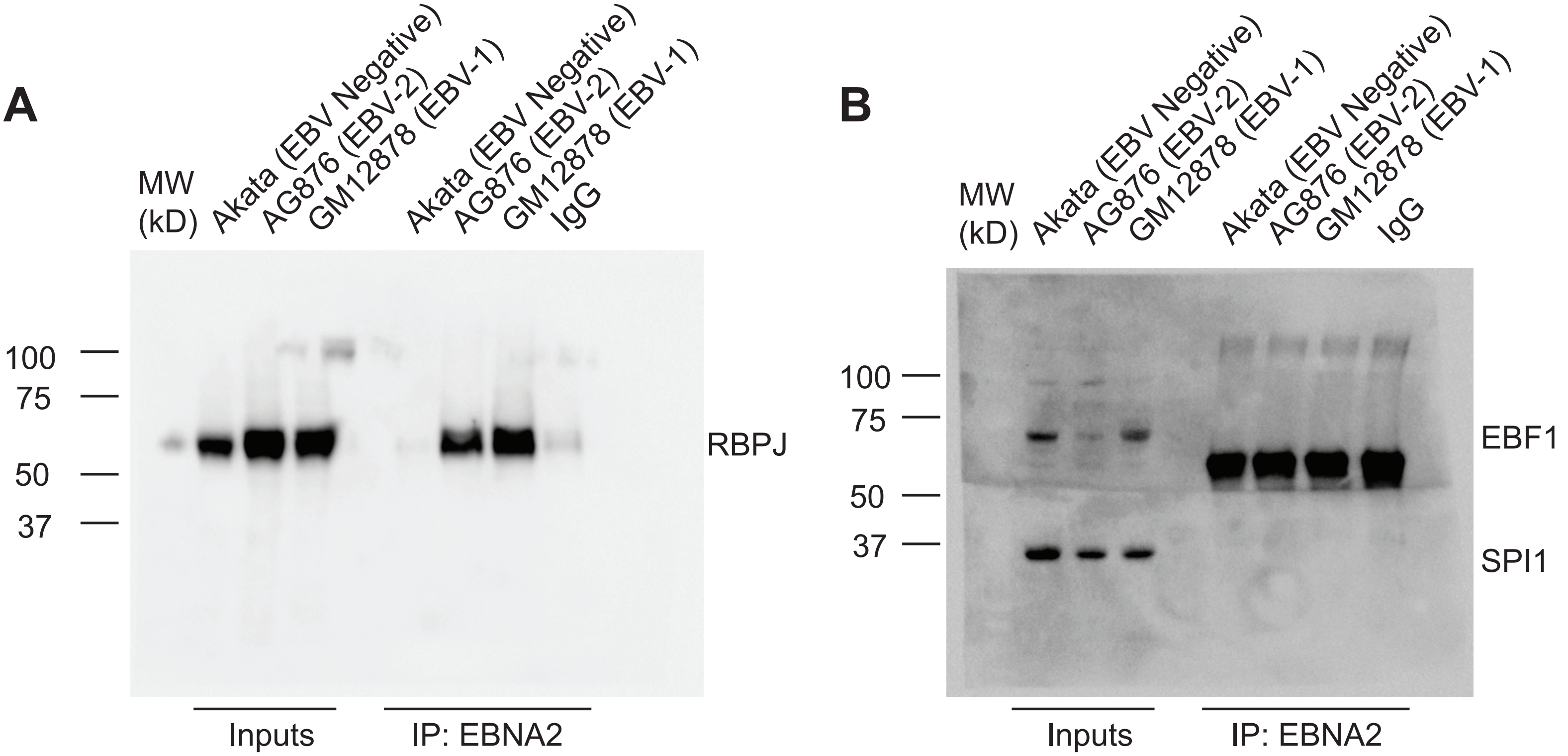


**Additional File 6: Supplemental Figure 6. EBNA2 forms protein complexes with RBPJ but not SPI1 or EBF1.** Co-Immunoprecipitation was performed on nuclear lysates in Akata (EBV negative), AG876 (EBV-2 infected), and GM12878 (EBV-1 infected) cell lines. Input represents the total nuclear lysate. Lysates were precipitated with EBNA2 then assayed by Western blot using RBPJ, EBF1, or SPI1 antibodies. (n = 3, one representative experiment is displayed).
