# Supplementary material for: Shared and distinct interactions of type 1 and type 2 Epstein-Barr Nuclear Antigen 2 with the human genome
Source: BMC Genomics. 2024 Mar 12;25:273. doi: 10.1186/s12864-024-10183-8 (PMC10935964; doi:10.1186/s12864-024-10183-8)
Supplement: Supplementary file 7 — Supplementary Material 7. [file 12864_2024_10183_MOESM7_ESM.pdf]

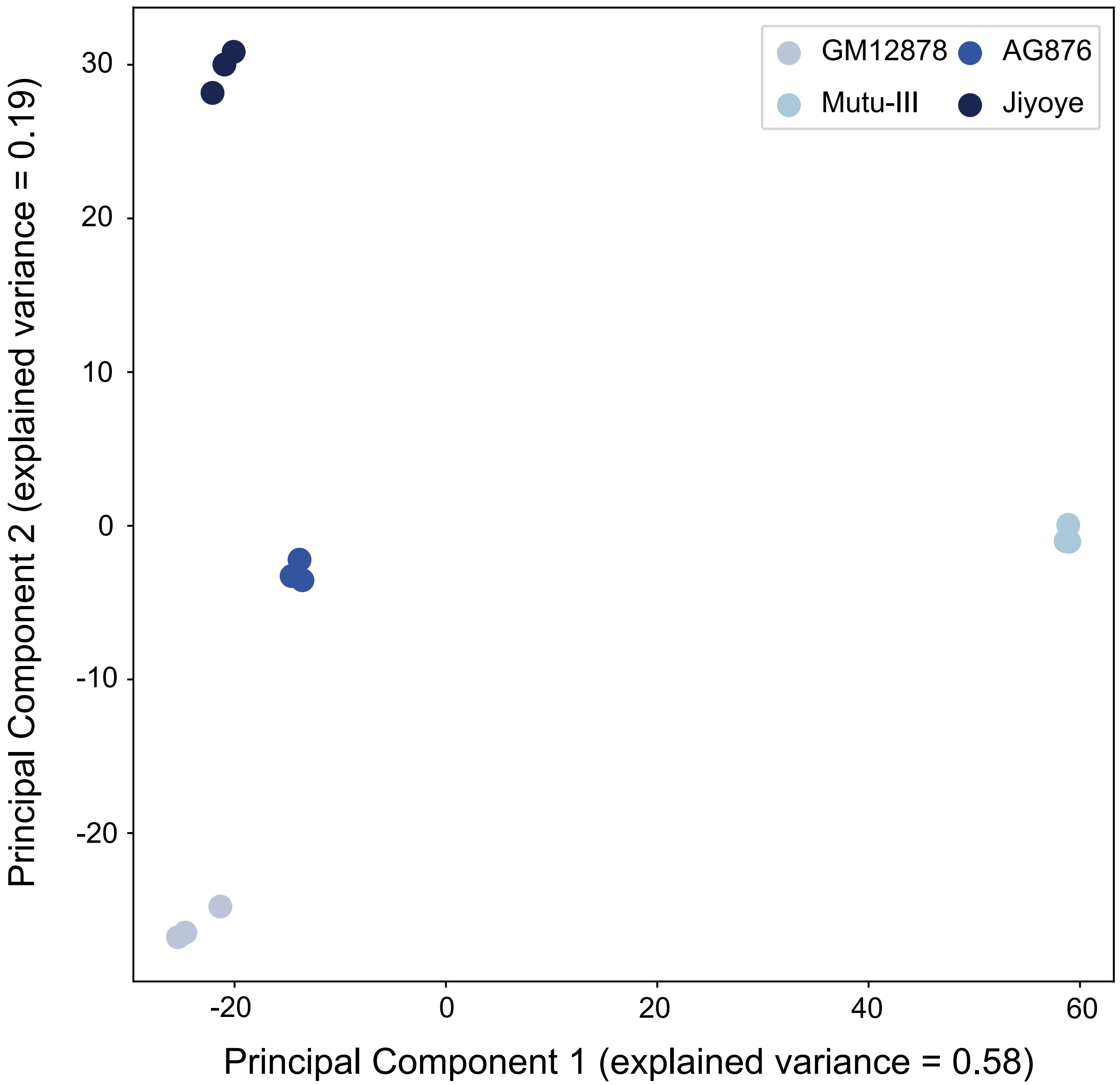

Additional File 7: Supplemental Figure 7. Comparison of RNA-seq datasets across type 1 and type 2 EBV B cell line replicates. Principal component analysis (PCA) on the 12 RNA-seq datasets used in this study. The publicly available Mutu-III RNA-seq dataset was sequenced single-end using a Poly(A) transcriptome library. AG876, Jiyoye, and GM12878 datasets (which were generated in this study) were sequenced paired-end using a whole transcriptome library. Experimental replicates cluster tightly together.
