# Supplementary material for: Shared and distinct interactions of type 1 and type 2 Epstein-Barr Nuclear Antigen 2 with the human genome
Source: BMC Genomics. 2024 Mar 12;25:273. doi: 10.1186/s12864-024-10183-8 (PMC10935964; doi:10.1186/s12864-024-10183-8)
Supplement: Supplementary file 8 — Supplementary Material 8. [file 12864_2024_10183_MOESM8_ESM.zip › Additional_File_8_Supplemental_Figure_8_REVISED.pdf]

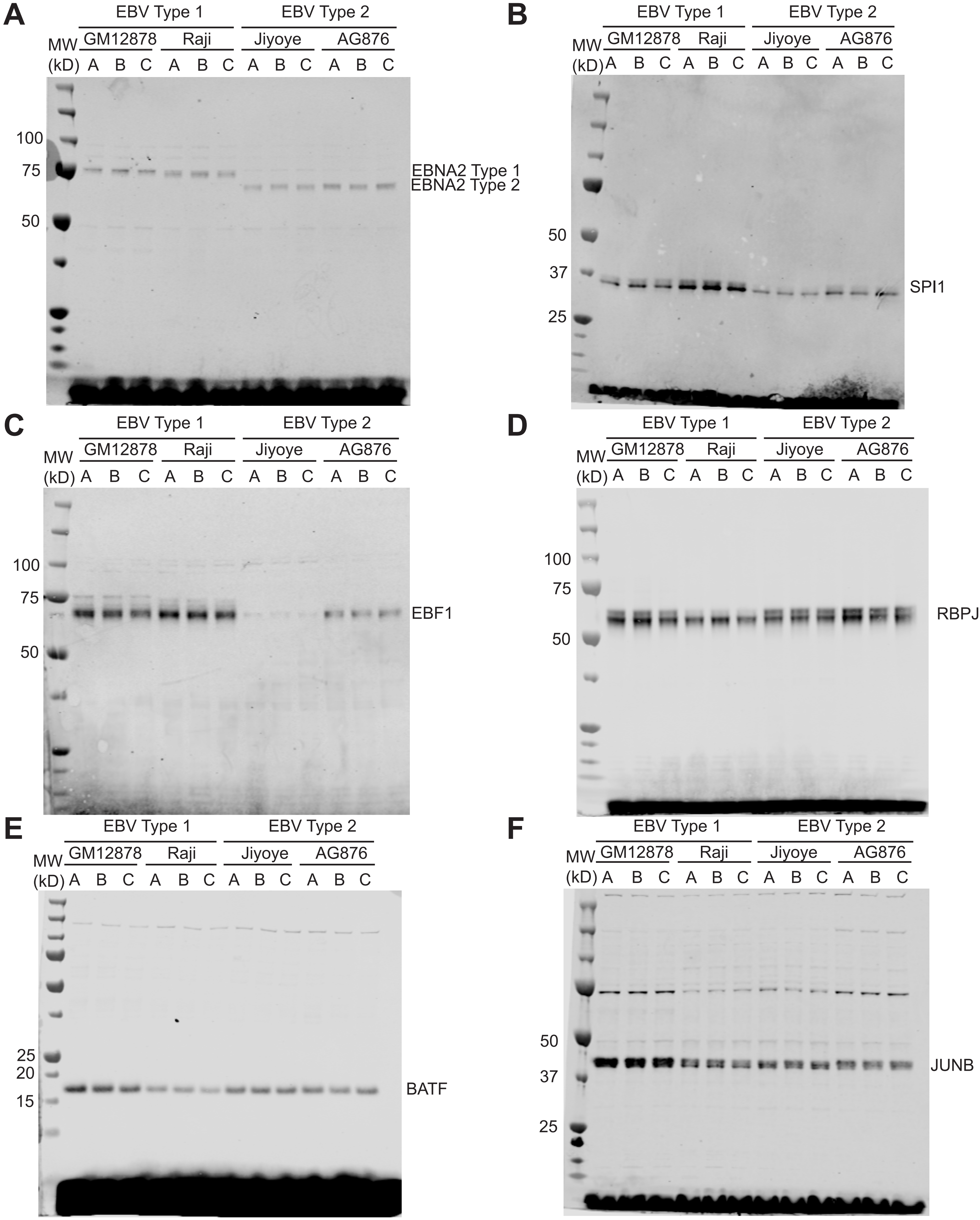

Additional File 8: Supplemental Figure 8. Proteomic analysis of EBNA2 and hTF expression levels across type 1 and type 2 EBV B cell lines. Western blots were performed on nuclear lysates for each cell line in biological triplicates. Quantification is provided in Table 2. Experiments were performed using type EBV-1 (GM12878 and Raji) and EBV-2 (AG876 and Jiyoye) transformed cell lines. Westerns were performed for: A) EBNA2 B) SPI1 C) EBF1 D) RBPJ E) BATF and F) JUNB.
