# Supplementary figures and images for: Shared and distinct interactions of type 1 and type 2 Epstein-Barr Nuclear Antigen 2 with the human genome
Source: BMC Genomics. 2024 Mar 12;25:273. doi: 10.1186/s12864-024-10183-8 (PMC10935964; doi:10.1186/s12864-024-10183-8)

## Additional File 8

Raw Images:

**EBF1**

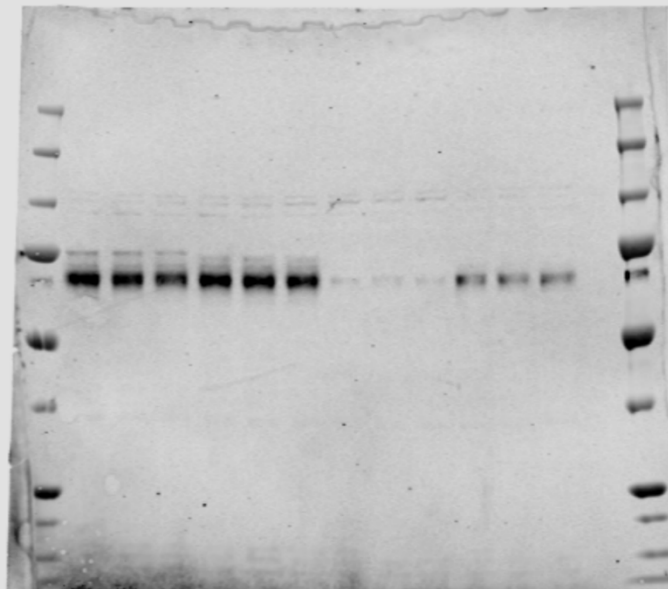

**SPI1**

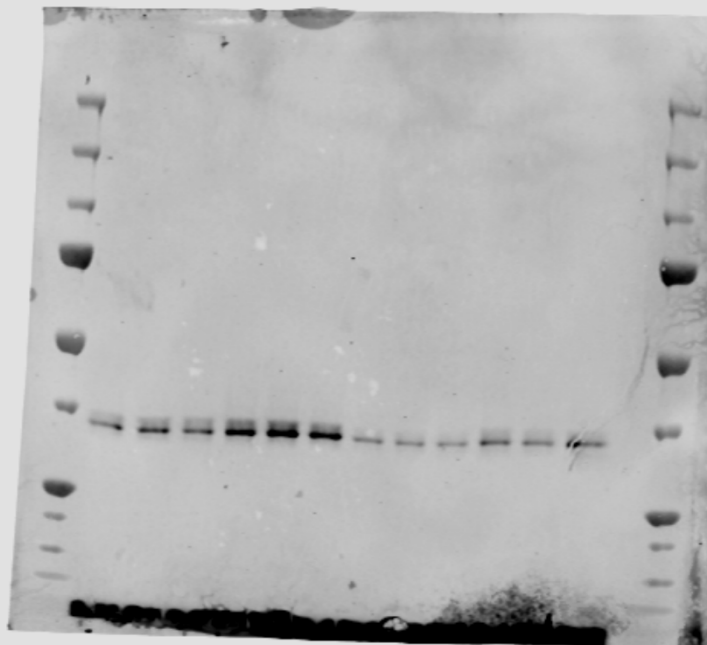

**JUNB**

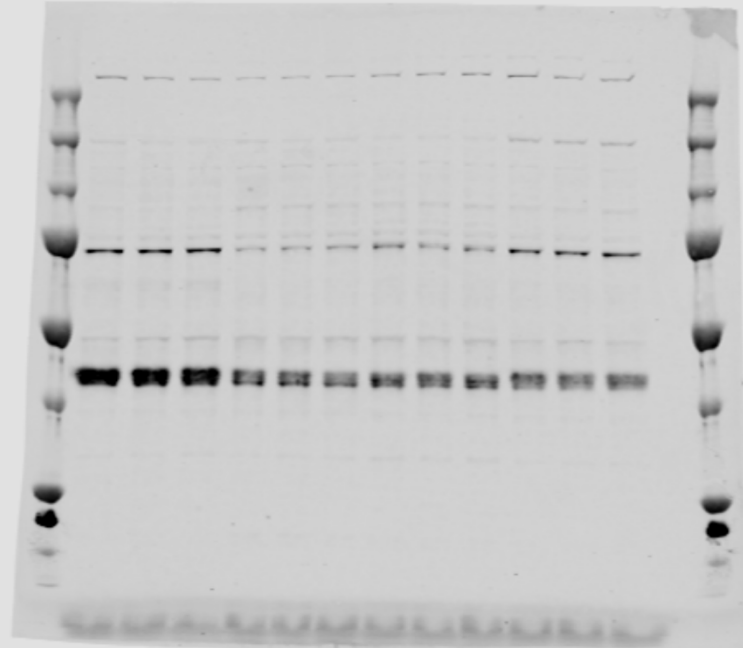

**EBNA2**

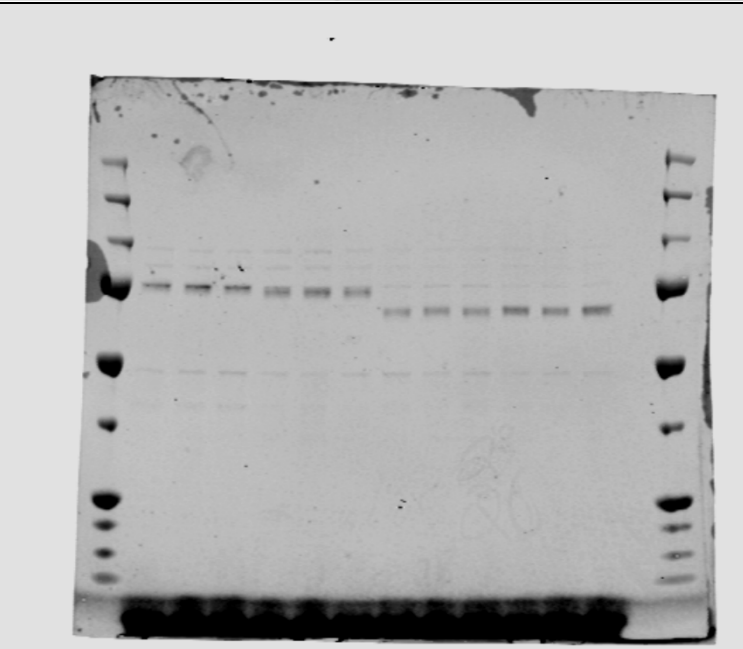

**BATF**

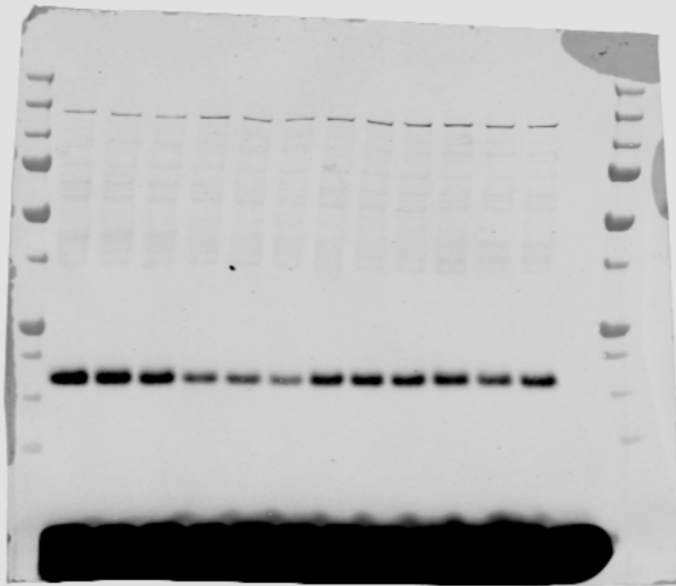

**RBPJ**

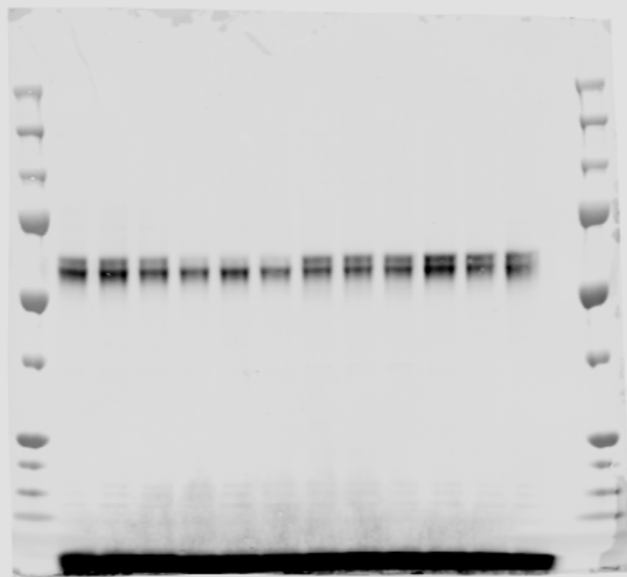

Final Image:

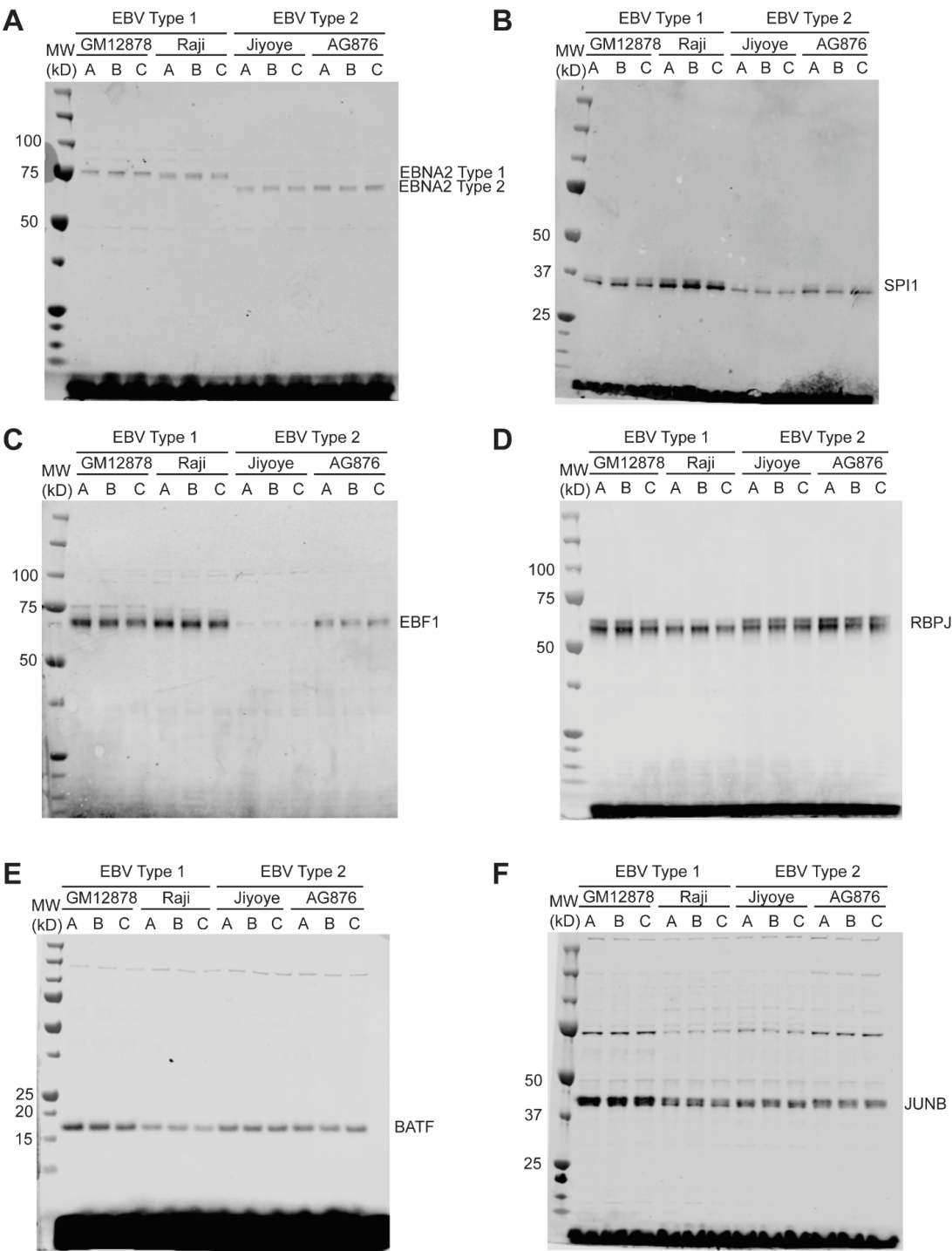

Supplement: Supplementary file 8 — Supplementary Material 8. [file 12864_2024_10183_MOESM8_ESM.zip › Additional File 8_with_raw_images.pdf]
