# Supplementary material for: Shared and distinct interactions of type 1 and type 2 Epstein-Barr Nuclear Antigen 2 with the human genome
Source: BMC Genomics. 2024 Mar 12;25:273. doi: 10.1186/s12864-024-10183-8 (PMC10935964; doi:10.1186/s12864-024-10183-8)
Supplement: Supplementary file 9 — Supplementary Material 9. [file 12864_2024_10183_MOESM9_ESM.pdf]

A: Predicted motif presence of EBF1

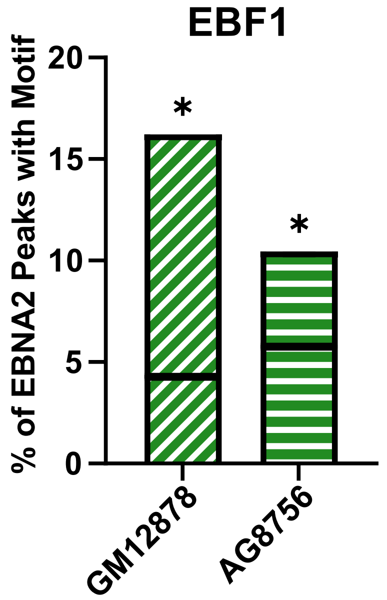

B: Validation: EBF1 ChIP-seq peak overlap

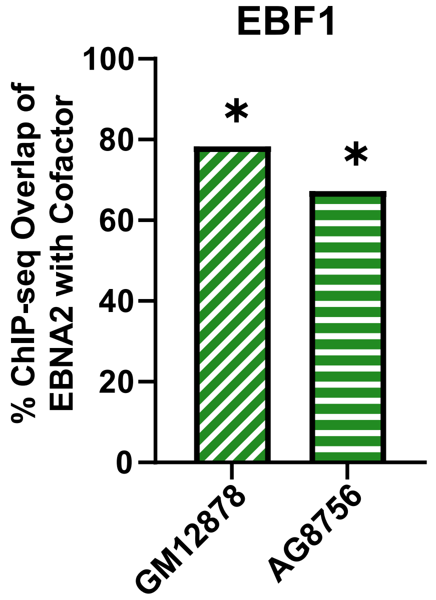

Additional File 9: Supplemental Figure 9. Identification of GM12878 and AG876 EBNA2 human cofactors. A) Frequency of occurrence of exemplary EBF1 motif in EBNA2 GM12878 (EBV-1) and AG876 (EBV-2) cell lines. The percent of peaks containing predicted binding sites for EBF1 is shown. Each bar represents percent foreground (i.e., the percent of actual peak DNA sequences containing a match to the motif). The horizonal black line within each bar depicts percent background (i.e., the percent of randomly selected genome sequences, matching GC content). Asterisks indicate significant motif enrichment (P<0.05), as calculated by HOMER. B) Experimental validation of predicted EBNA2 co-occupancy with EBF1 in GM12878 (EBV-1) and AG876 (EBV-2) cell lines. Co-occupancy was assessed by EBF1 and EBNA2 ChIP-seq peak overlap. For each bar, the percent of each EBF1 peak set overlapping each EBNA2 ChIP-seq peak category is shown. Datasets with significant overlap between EBNA2 peak sets and EBF1 peaks (as calculated by RELI) are indicated with asterisks (P<0.05).
