# Supplementary material for: Shared and distinct interactions of type 1 and type 2 Epstein-Barr Nuclear Antigen 2 with the human genome
Source: BMC Genomics. 2024 Mar 12;25:273. doi: 10.1186/s12864-024-10183-8 (PMC10935964; doi:10.1186/s12864-024-10183-8)
Supplement: Supplementary file 10 — Supplementary Material 10. [file 12864_2024_10183_MOESM10_ESM.zip › Additional_File_10_Supplemental_Figure_10_REVISED.pdf]

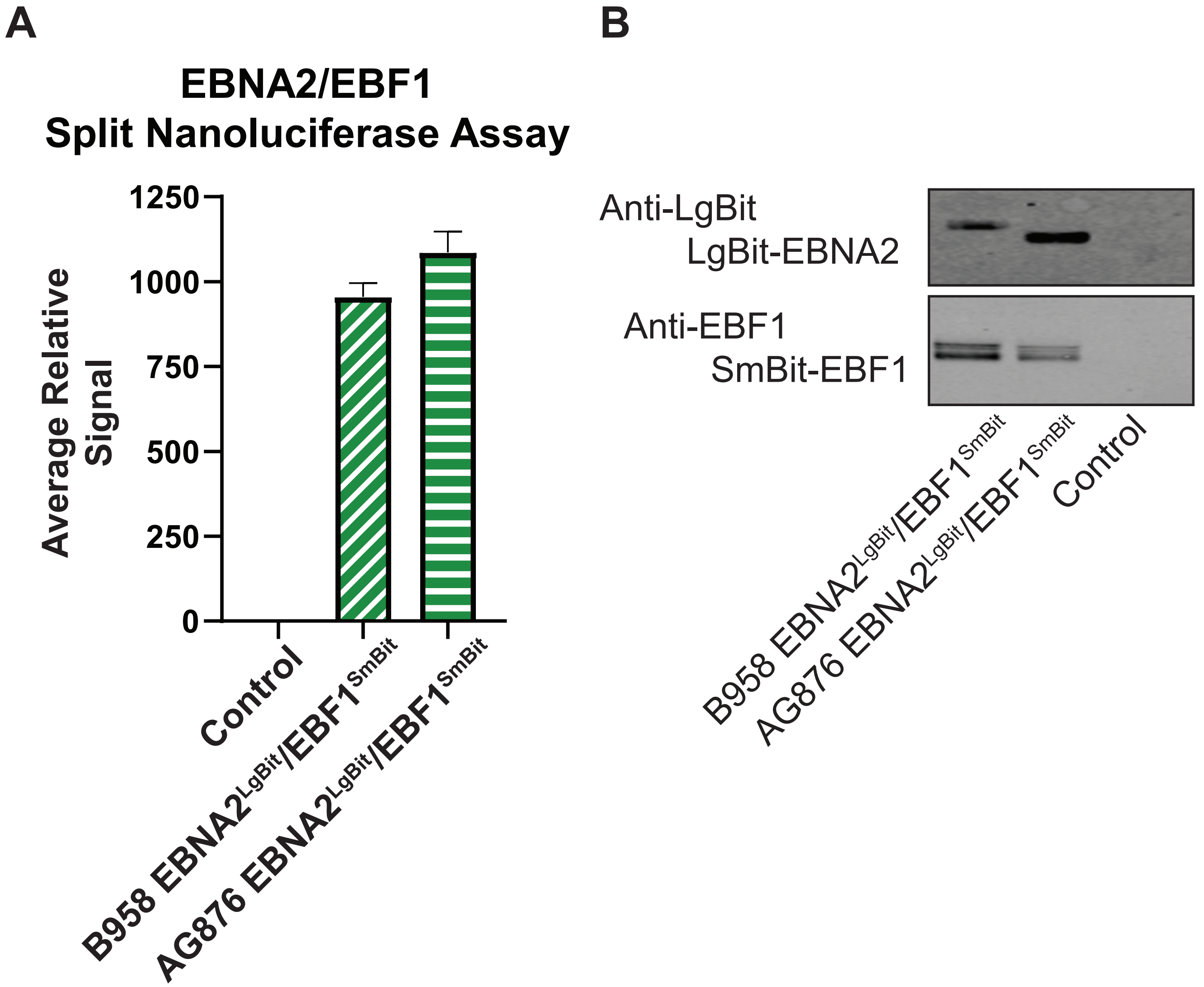

Additional File 10: Supplemental Figure 10. EBNA2 type 1 and type 2 both colocalize with EBF1. A) The average relative luminescence activity of split nanoluciferase. Equivalent levels of SmBit-EBF1 were transfected with either LgBit-EBNA2 type 1 or LgBit-EBNA2 type 2 into HEK-293 Cells. Luminescence activity was measured showing close proximity between EBNA2 and EBF1. B) Proteomic Analysis of LgBit-EBNA2 and SmBit-EBF1 fusion proteins in HEK-293 Cells shows relatively equal protein expression of the molecules across the two experimental conditions. The blot images were cropped for clarity. A version of the full image was provided to BMC Genomics.
