# Supplementary material for: Shared and distinct interactions of type 1 and type 2 Epstein-Barr Nuclear Antigen 2 with the human genome
Source: BMC Genomics. 2024 Mar 12;25:273. doi: 10.1186/s12864-024-10183-8 (PMC10935964; doi:10.1186/s12864-024-10183-8)

Additional Figure 10

Raw Images:

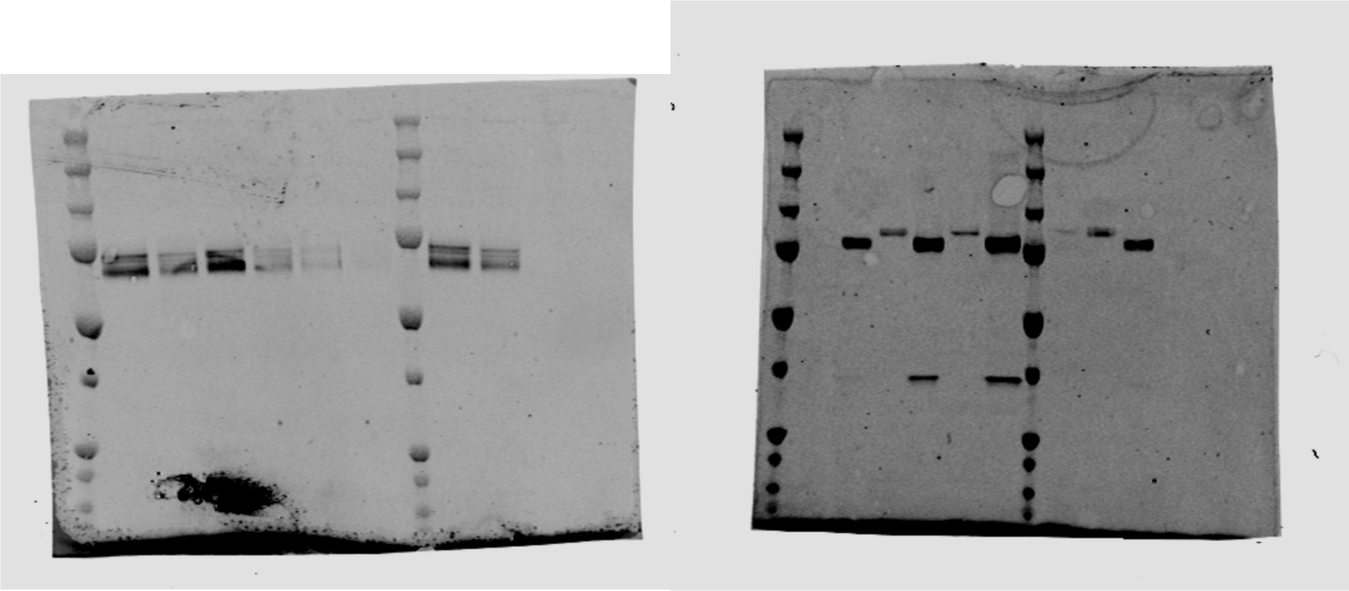

Labelled Raw Images:

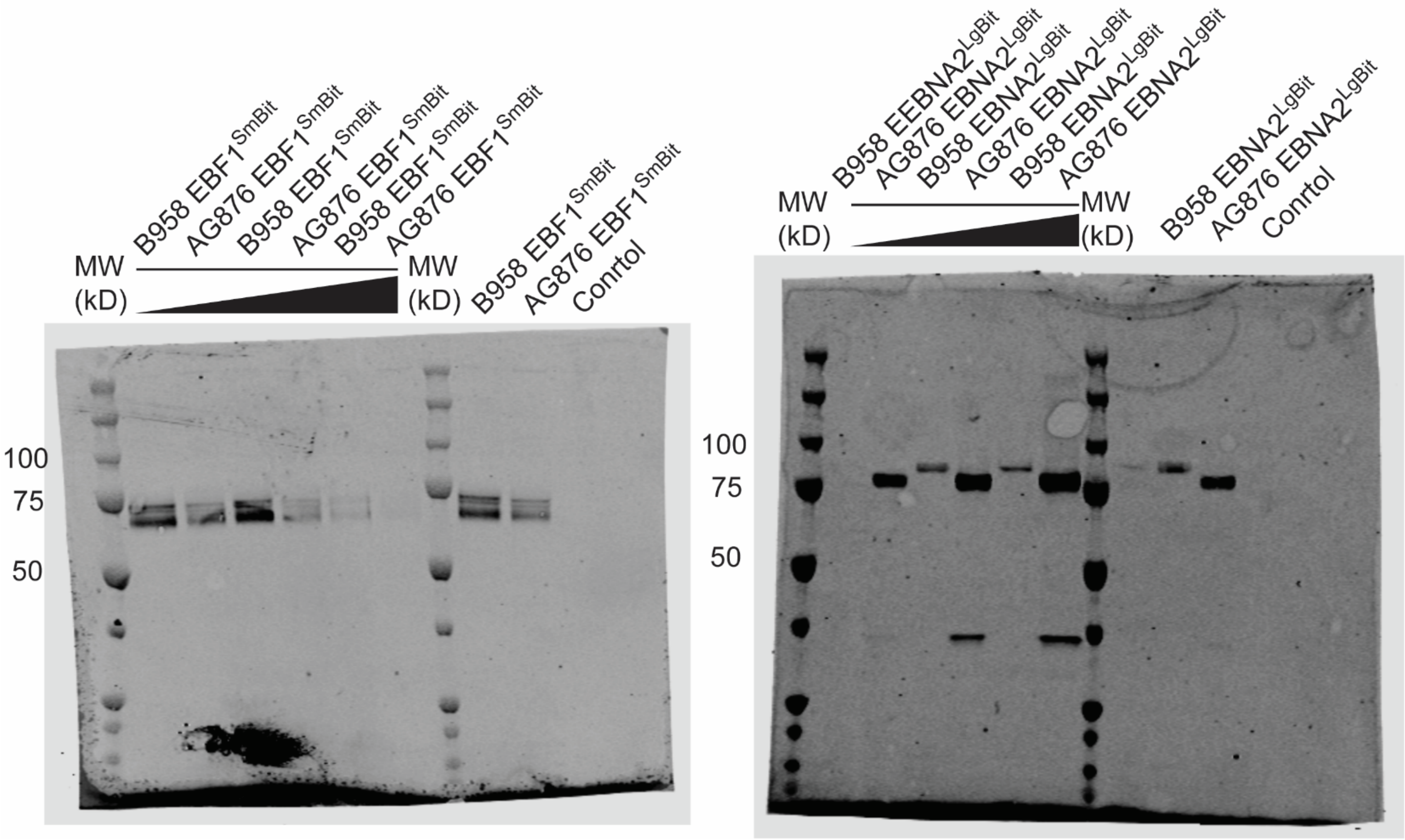

Sections used for Final Paper:

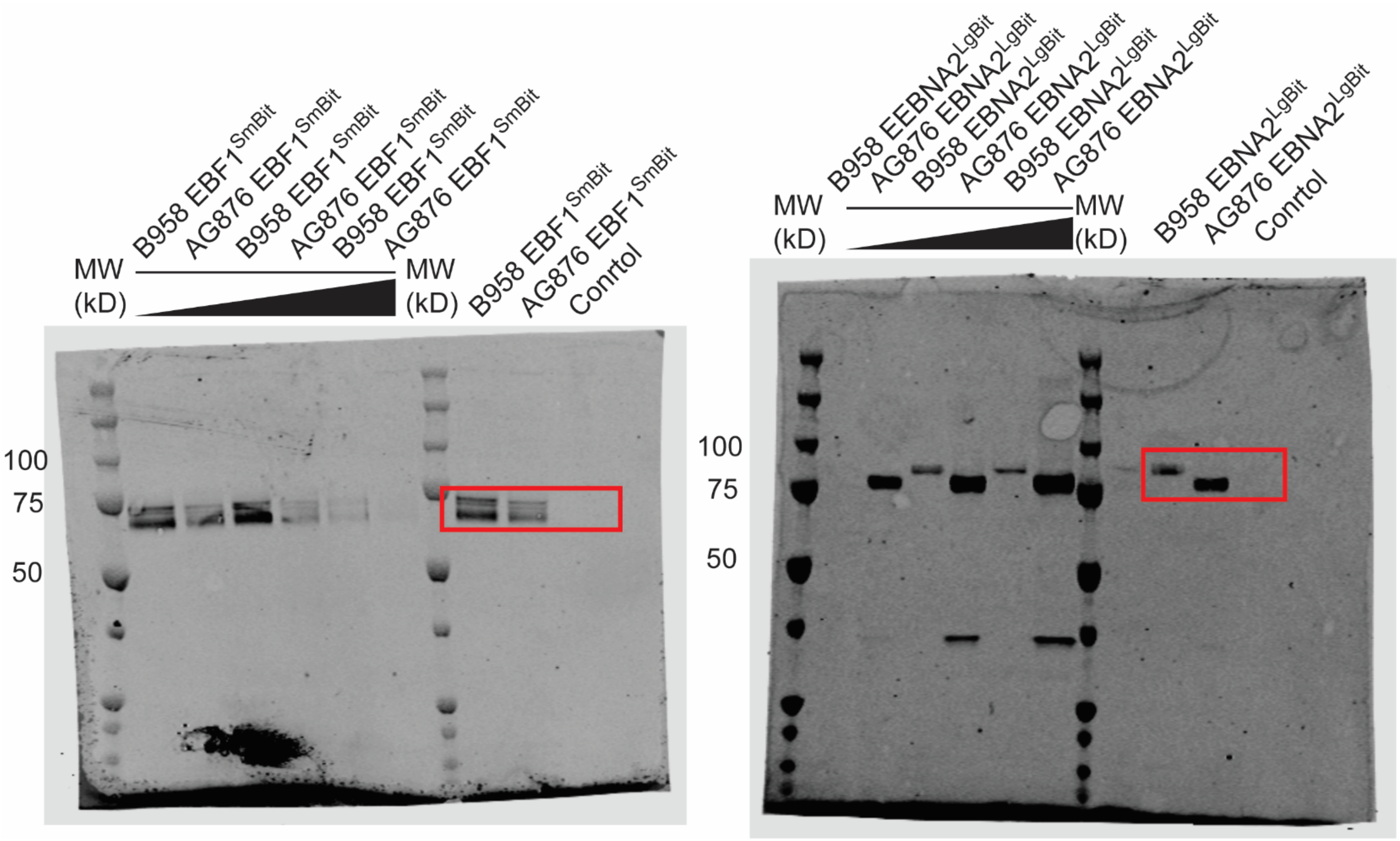

Final Image for Paper:

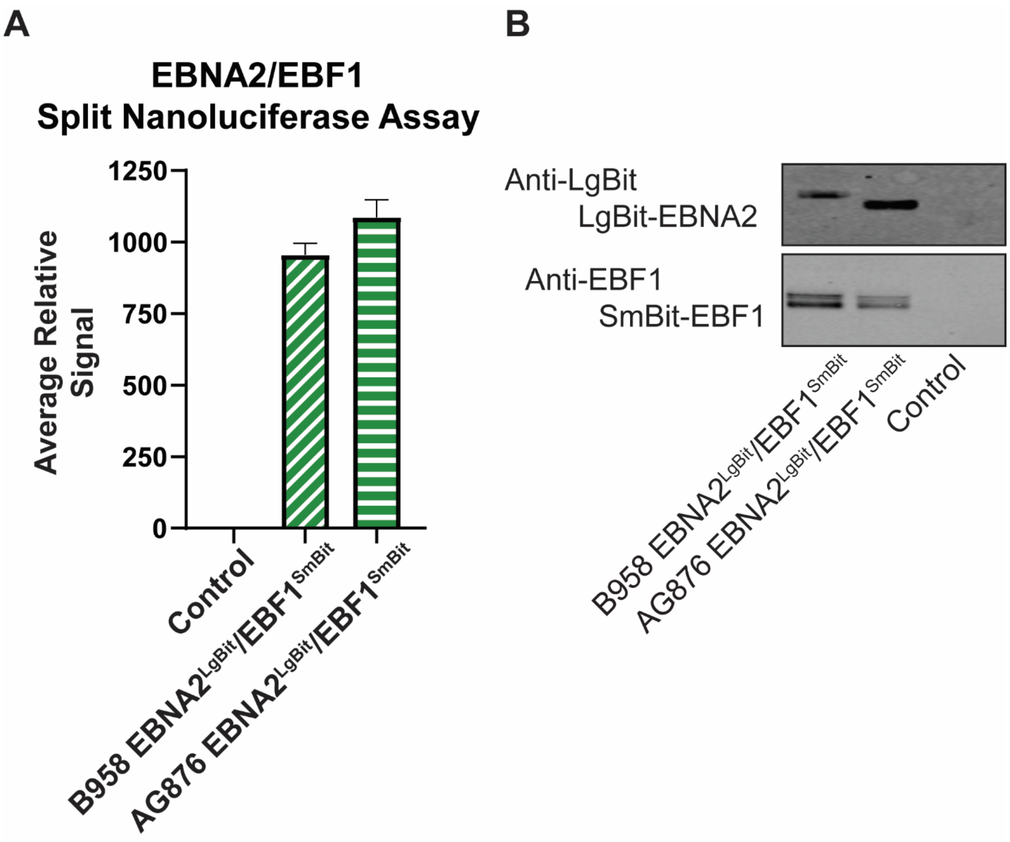

Supplement: Supplementary file 10 — Supplementary Material 10. [file 12864_2024_10183_MOESM10_ESM.zip › Additional_File_10_with_raw_images.pdf]
