# Supplementary material for: Shared and distinct interactions of type 1 and type 2 Epstein-Barr Nuclear Antigen 2 with the human genome
Source: BMC Genomics. 2024 Mar 12;25:273. doi: 10.1186/s12864-024-10183-8 (PMC10935964; doi:10.1186/s12864-024-10183-8)
Supplement: Supplementary file 11 — Supplementary Material 11. [file 12864_2024_10183_MOESM11_ESM.pdf]

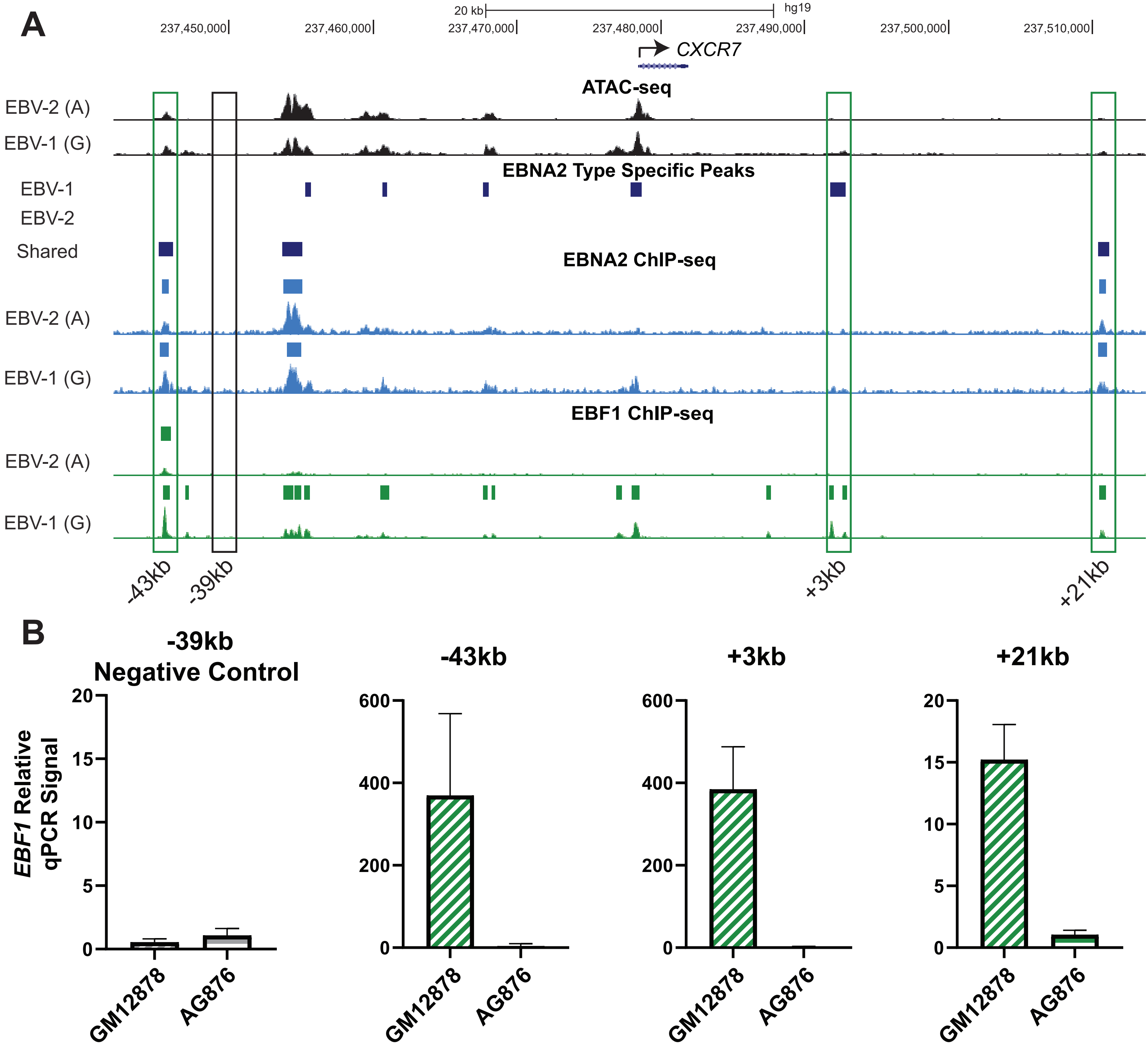

Additional File 11: Supplemental Figure 11. EBF1 ChIP-qPCR analysis of EBNA2 ChIP-seq peaks in the CXCR7 locus. A) Genome Browser track showing chromatin accessibility and EBNA2 & EBF1 ChIP-seq signal at the CXCR7 locus. Range: ATAC-seq (0 to 5); EBNA2 ChIP-seq (0 to 1.5); and EBF1 ChIP-seq (0 to 4). A = AG876; G = GM12878 B) Chromatin immunoprecipitation of EBF1 at the CXCR7 locus (chr2:237,441,986-237,513,677) was normalized to the cycle threshold of the AG876 immunoprecipitation. The positions of each qPCR plot are relative to the transcriptional start site of CXCR7. -39kb was selected as a negative control. The mean of three technical replicates is plotted; error bars are SD.
