# Supplementary material for: Shared and distinct interactions of type 1 and type 2 Epstein-Barr Nuclear Antigen 2 with the human genome
Source: BMC Genomics. 2024 Mar 12;25:273. doi: 10.1186/s12864-024-10183-8 (PMC10935964; doi:10.1186/s12864-024-10183-8)
Supplement: Supplementary file 19 — Supplementary Material 19. [file 12864_2024_10183_MOESM19_ESM.zip › index.html]

Shared and Distinct Interactions of Type 1 and Type 2 Epstein-Barr Nuclear Antigen 2 with the Human Genome


# Shared and Distinct Interactions of Type 1 and Type 2 Epstein-Barr Nuclear Antigen 2 with the Human Genome

This document contains the QC Summary Report for all ChIP, ATAC, and RNA-Seq dataset used.

Comments, additions, or subtractions should be addressed to the corresponding authors:

- Leah Kottyan
- Matt Weirauch

The `.html` files in this directory correspond with the tabs of the spreadsheet `Additional_File_10.xlsx`, included with the manuscript. A ZIP archive is containing all the reports is available. They can be viewed with any web browser.

The summary QC report for RNA-Seq data was created with MultiQC.

## QC-ChIP-Summary

- AG876.BATF.QC.html
- AG876.EBF1.QC.html
- AG876.EBNA2.QC.html
- AG876.JUNB.QC.html
- AG876.RBPJ.QC.html
- AG876.SPI1.QC.html
- GM12878.BATF.QC.html
- GM12878.EBF1.QC.html
- GM12878.EBNA2.QC.html
- GM12878.JUNB.QC.html
- GM12878.RBPJ.QC.html
- GM12878.SPI1.QC.html
- Jiyoye.BATF.QC.html
- Jiyoye.EBNA2.QC.html
- Jiyoye.JUNB.QC.html
- Jiyoye.RBPJ.QC.html
- Jiyoye.SPI1.QC.html
- LCL.EBF1.QC.html
- MutuIII.EBNA2.QC.html

## QC-ATAC-Summary

- AG876.ATAC.QC.html
- GM12878.ATAC.QC.html
- Jiyoye.ATAC.QC.html
- MutuIII.ATAC.QC.html

## QC-RNA-Summary

- RNA\_MultiQC\_Report.html
